# Supplementary figures and images for: High throughput screening and identification of coagulopathic snake venom proteins and peptides using nanofractionation and proteomics approaches
Source: PLoS Negl Trop Dis. 2020 Apr 1;14(4):e0007802. doi: 10.1371/journal.pntd.0007802 (PMC7153897; doi:10.1371/journal.pntd.0007802)

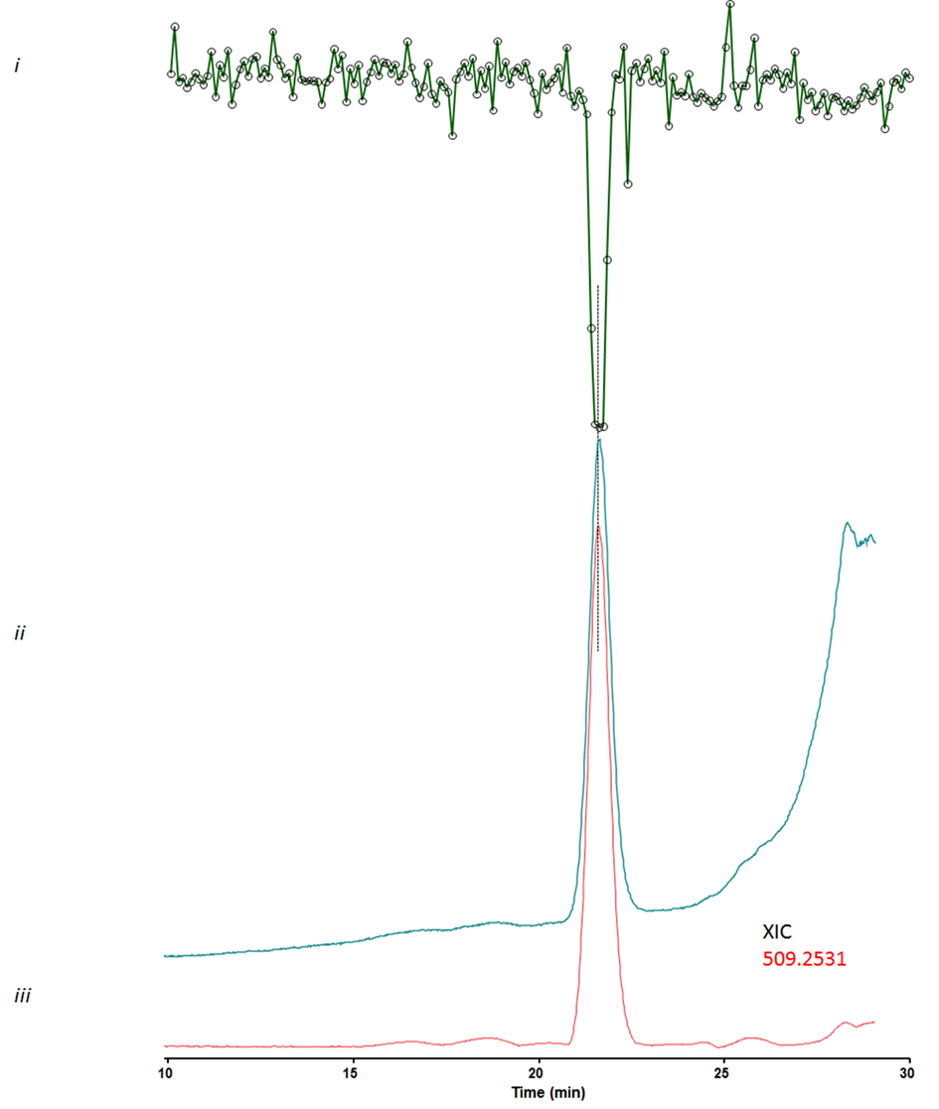

Supplement: S1 Fig — (TIF) [file pntd.0007802.s001.tif]

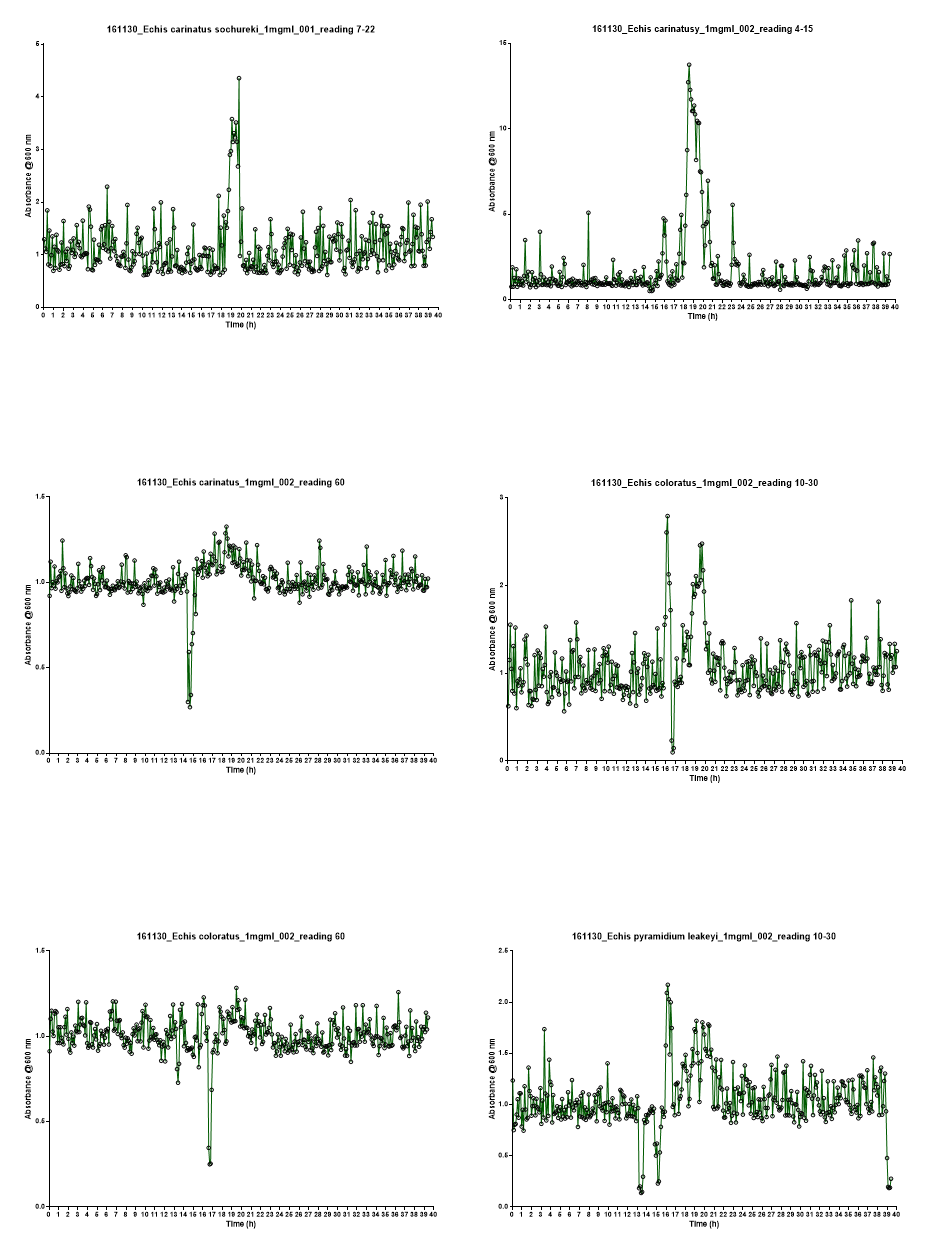

Supplement: S2 Fig — (ZIP) [file pntd.0007802.s002.zip › S1 fig 2.1 Initial screening results of all 20 species included in the study.tif]

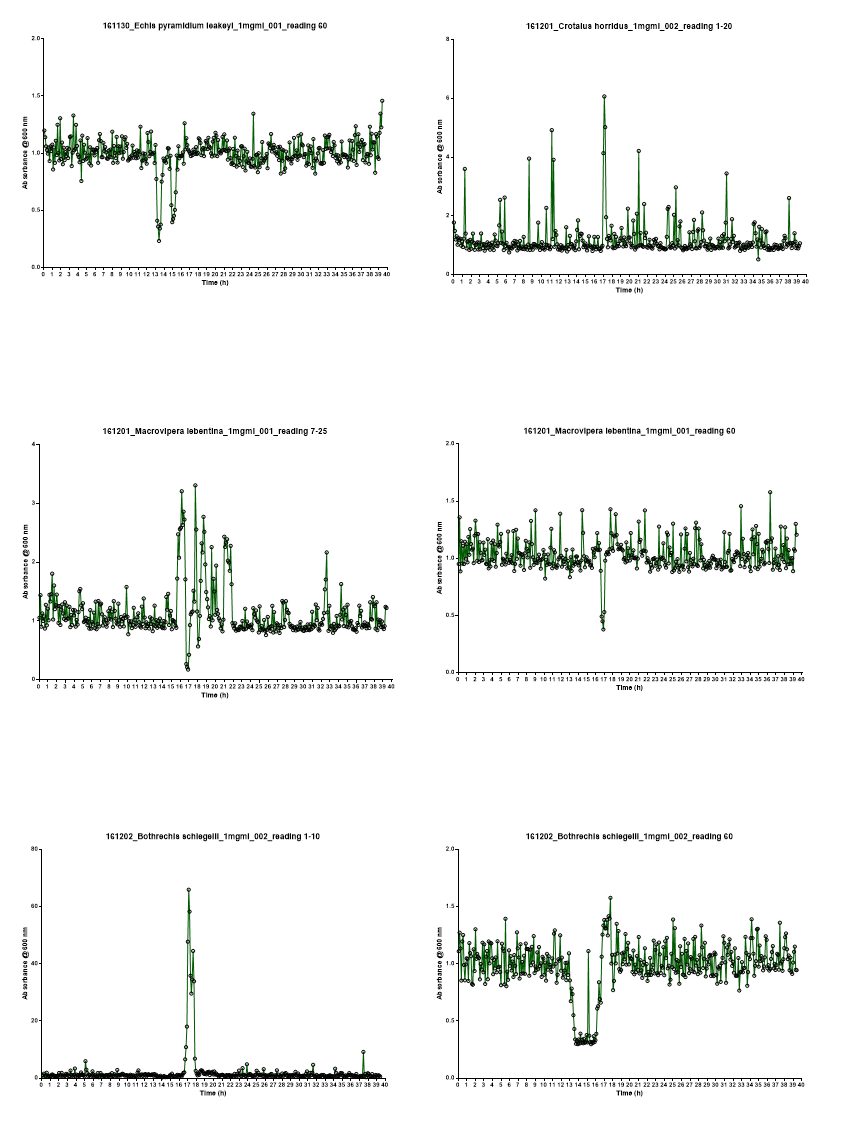

Supplement: S2 Fig — (ZIP) [file pntd.0007802.s002.zip › S1 fig 2.2 Initial screening results of all 20 species included in the study.tif]

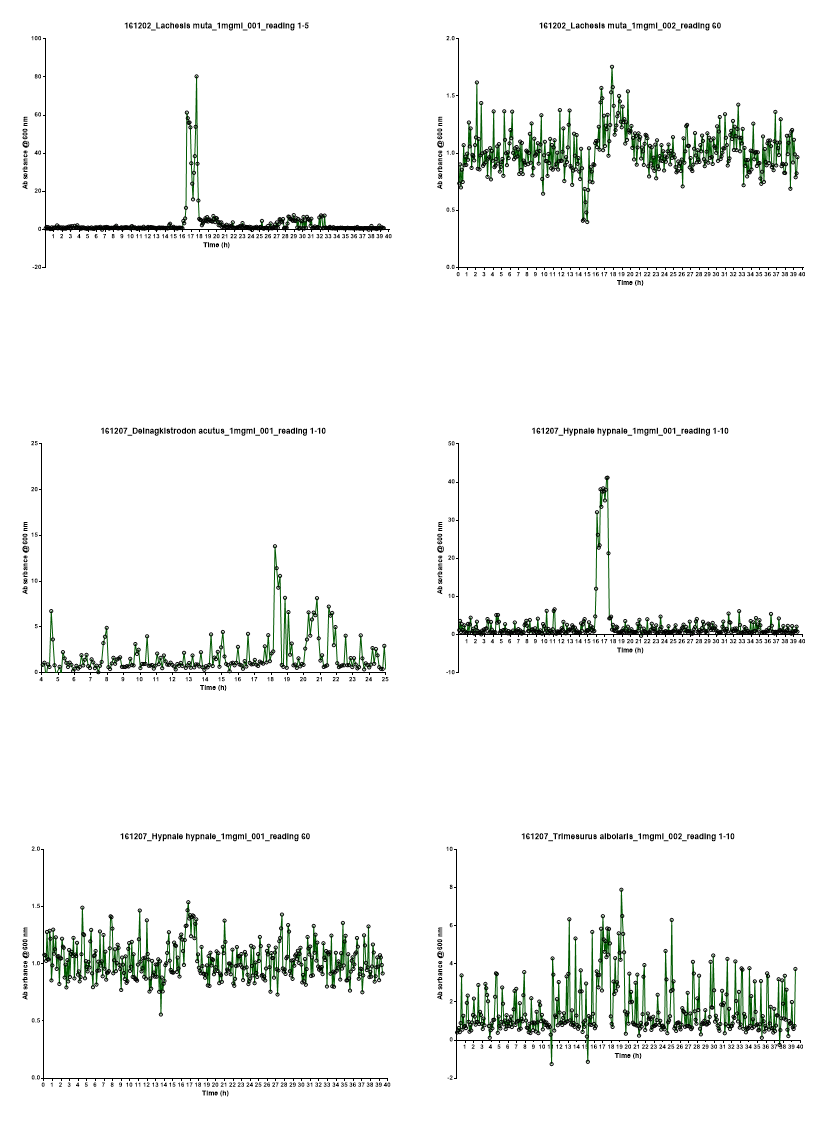

Supplement: S2 Fig — (ZIP) [file pntd.0007802.s002.zip › S1 fig 2.3 Initial screening results of all 20 species included in the study.tif]

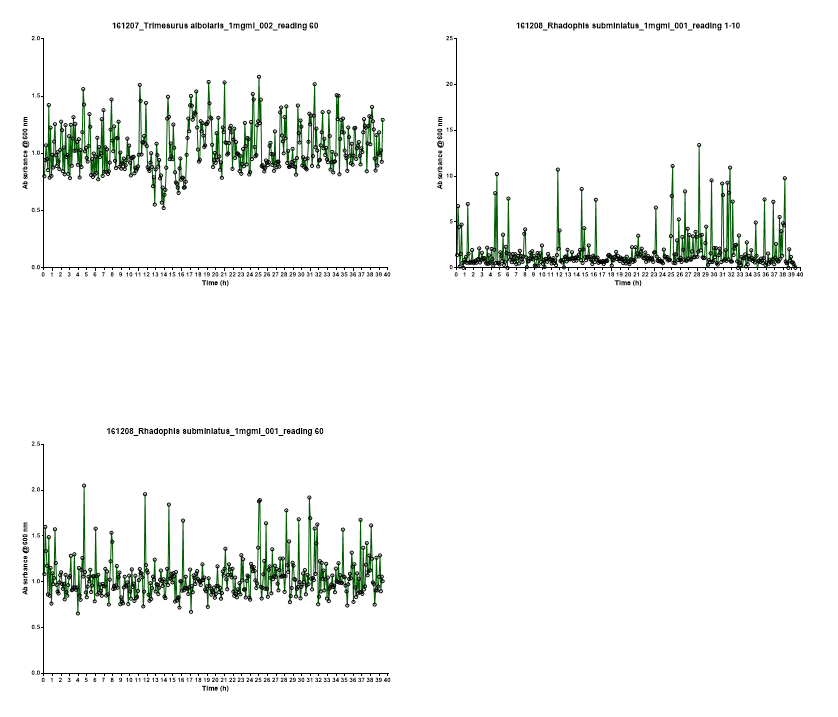

Supplement: S2 Fig — (ZIP) [file pntd.0007802.s002.zip › S1 fig 2.4 Initial screening results of all 20 species included in the study.tif]

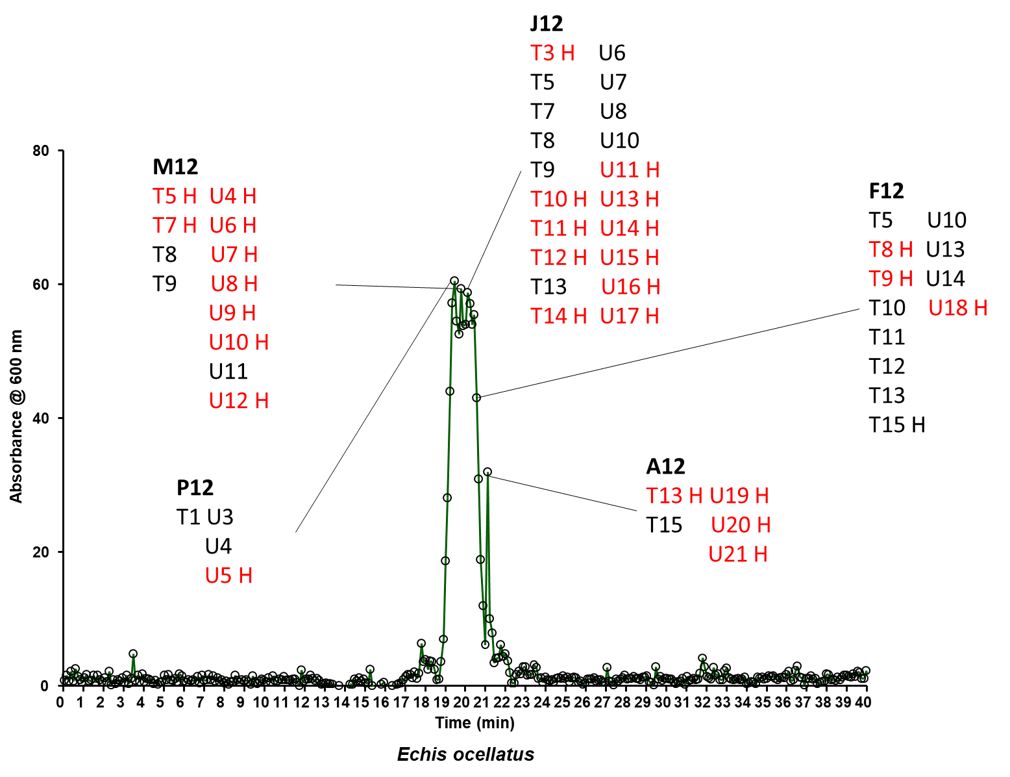

Supplement: S3 Fig — (ZIP) [file pntd.0007802.s003.zip › S1 Fig 3.1 Toxin IDs from the specific and non-specific databases.tif]

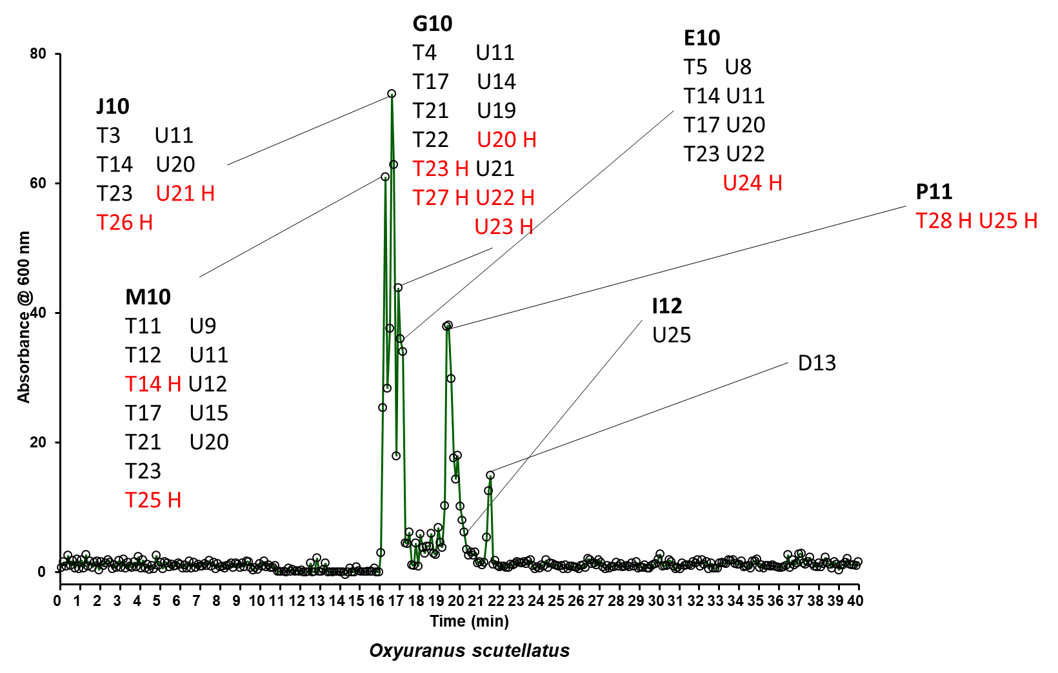

Supplement: S3 Fig — (ZIP) [file pntd.0007802.s003.zip › S1 Fig 3.10 Toxin IDs from the specific and non-specific databases.tif]

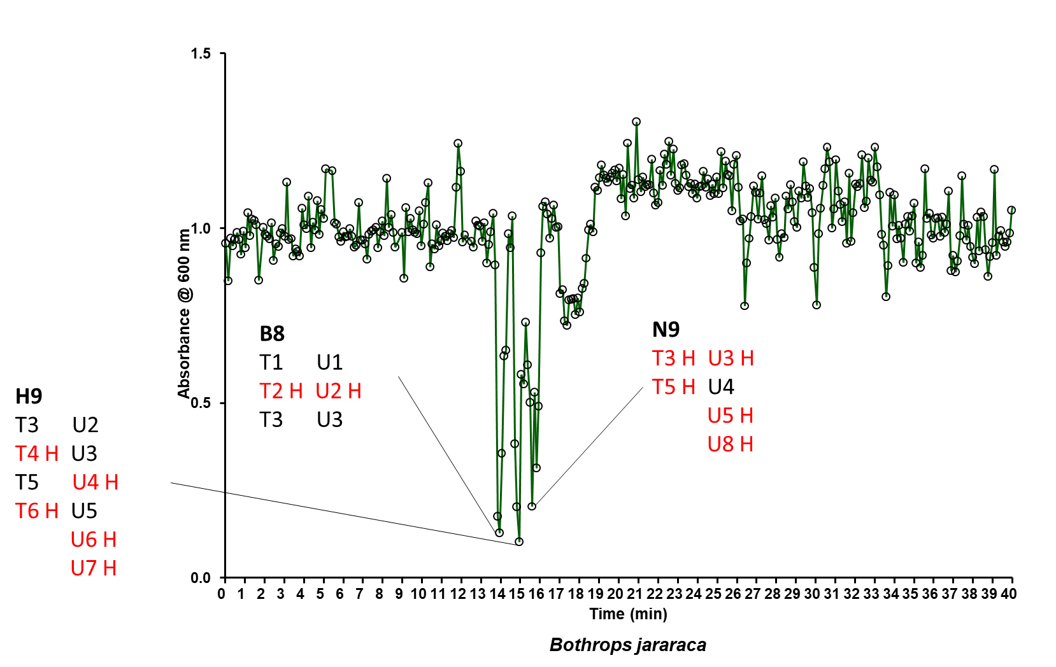

Supplement: S3 Fig — (ZIP) [file pntd.0007802.s003.zip › S1 Fig 3.11 Toxin IDs from the specific and non-specific databases.tif]

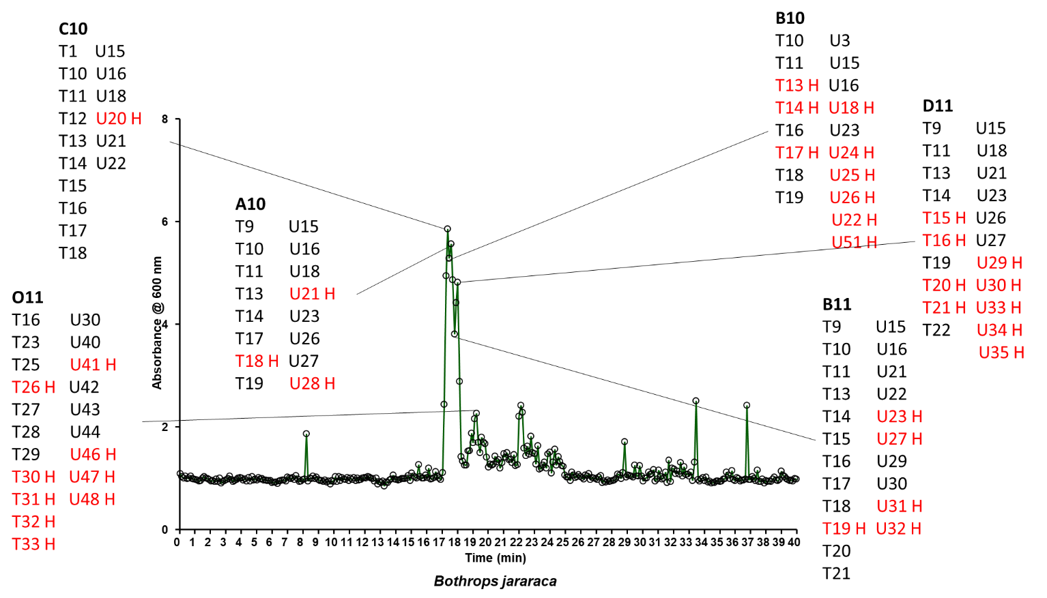

Supplement: S3 Fig — (ZIP) [file pntd.0007802.s003.zip › S1 Fig 3.12 Toxin IDs from the specific and non-specific databases.tif]

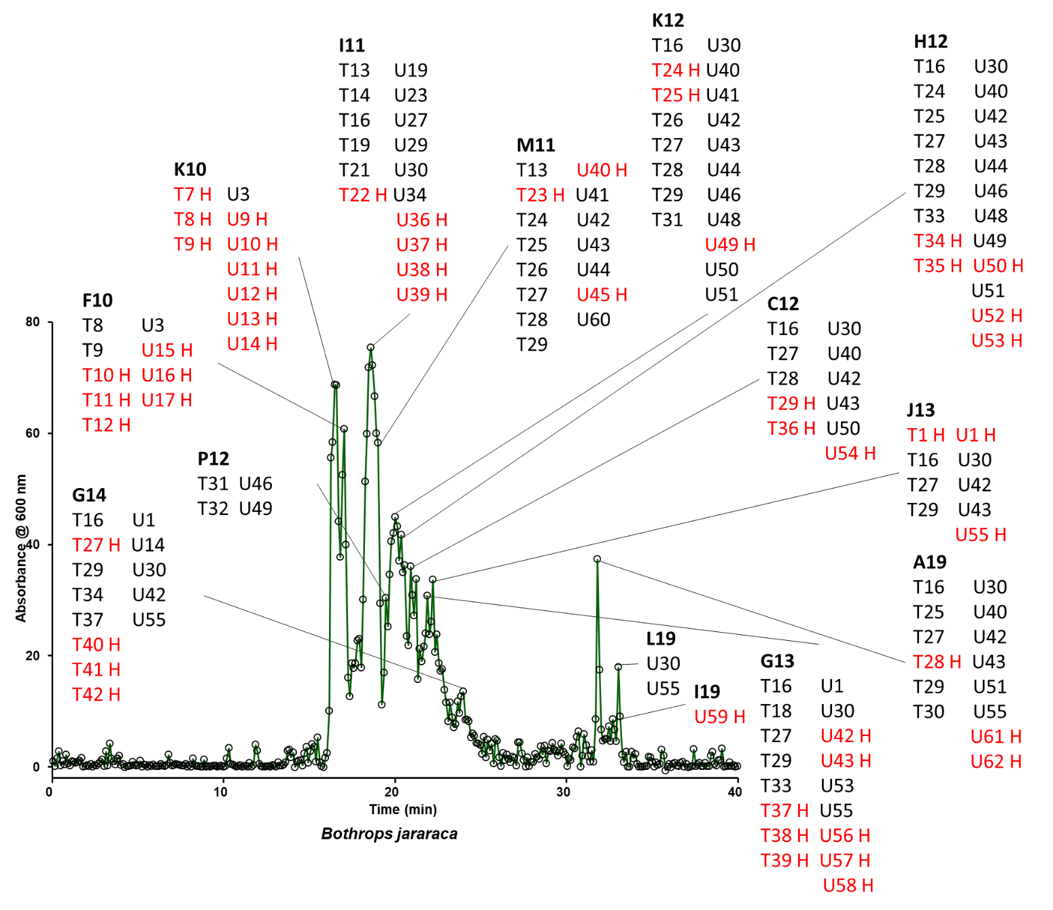

Supplement: S3 Fig — (ZIP) [file pntd.0007802.s003.zip › S1 Fig 3.13 Toxin IDs from the specific and non-specific databases.tif]

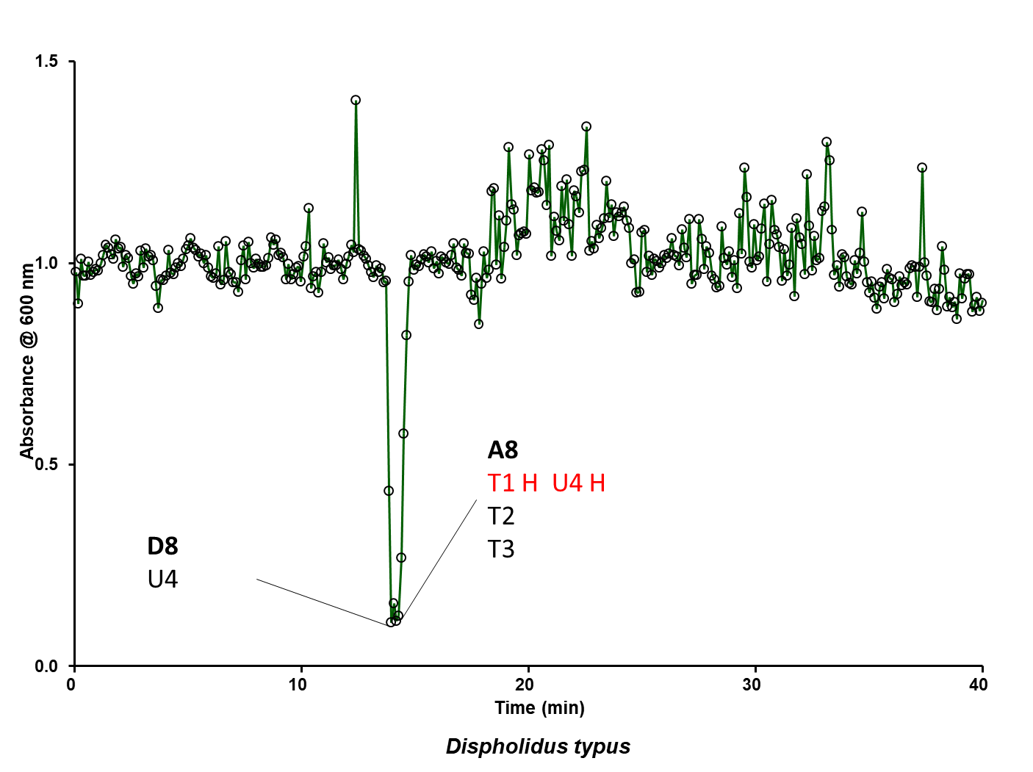

Supplement: S3 Fig — (ZIP) [file pntd.0007802.s003.zip › S1 Fig 3.14 Toxin IDs from the specific and non-specific databases.tif]

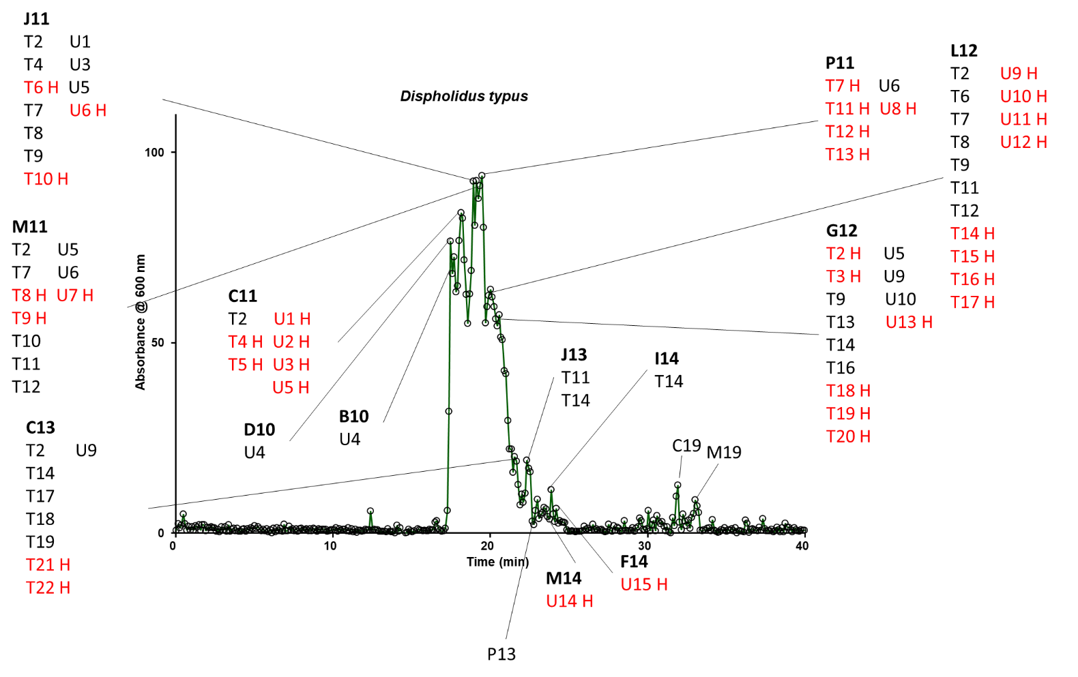

Supplement: S3 Fig — (ZIP) [file pntd.0007802.s003.zip › S1 Fig 3.15 Toxin IDs from the specific and non-specific databases.tif]

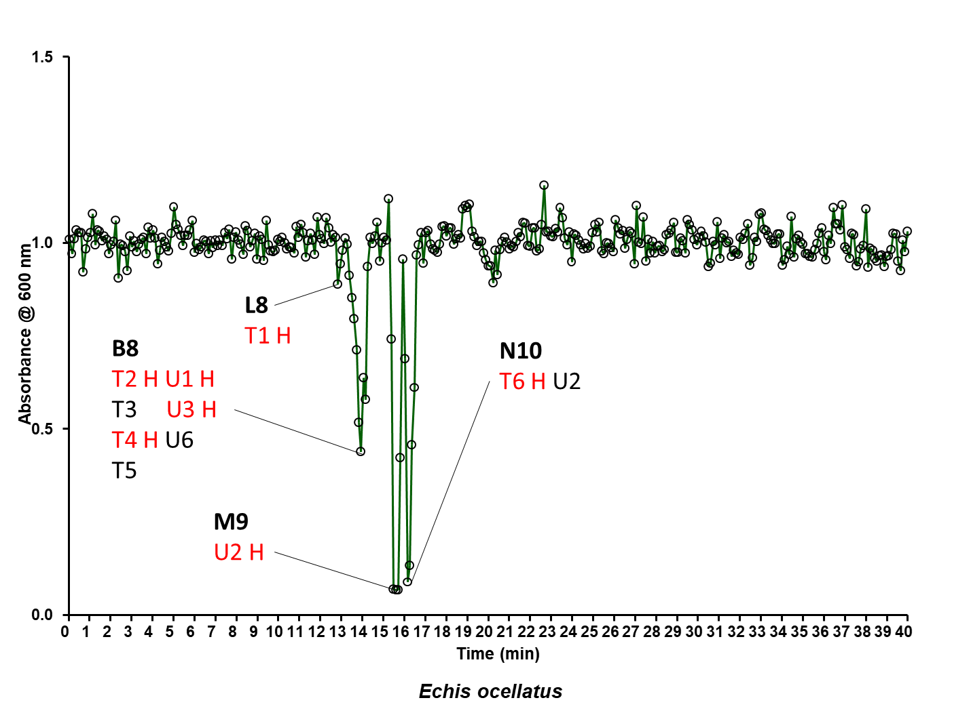

Supplement: S3 Fig — (ZIP) [file pntd.0007802.s003.zip › S1 Fig 3.2 Toxin IDs from the specific and non-specific databases.tif]

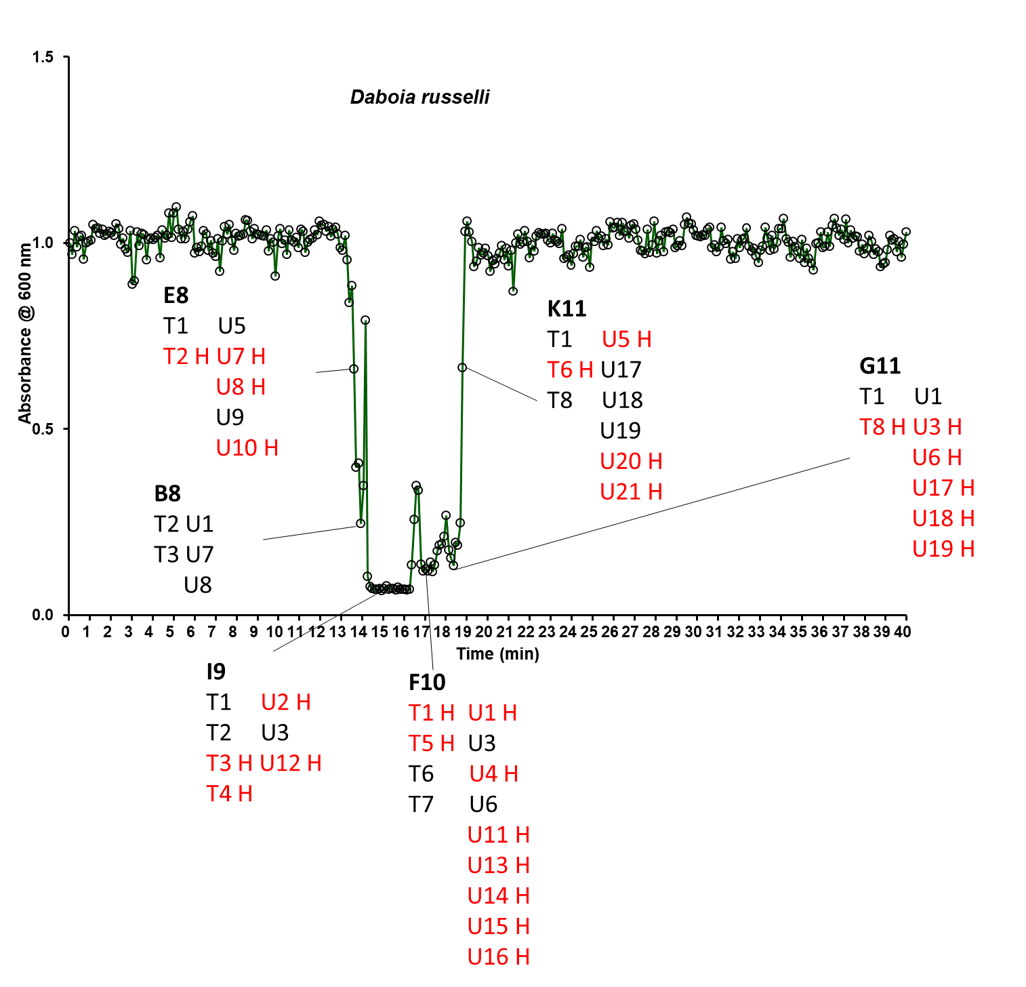

Supplement: S3 Fig — (ZIP) [file pntd.0007802.s003.zip › S1 Fig 3.3 Toxin IDs from the specific and non-specific databases.tif]

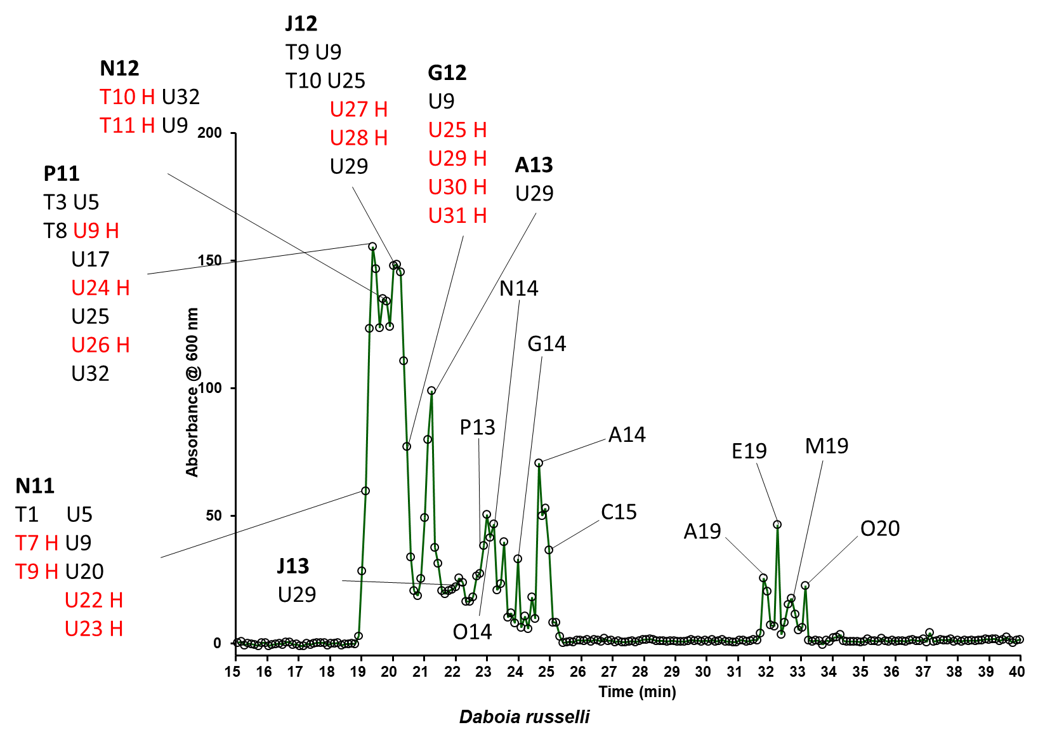

Supplement: S3 Fig — (ZIP) [file pntd.0007802.s003.zip › S1 Fig 3.4 Toxin IDs from the specific and non-specific databases.tif]

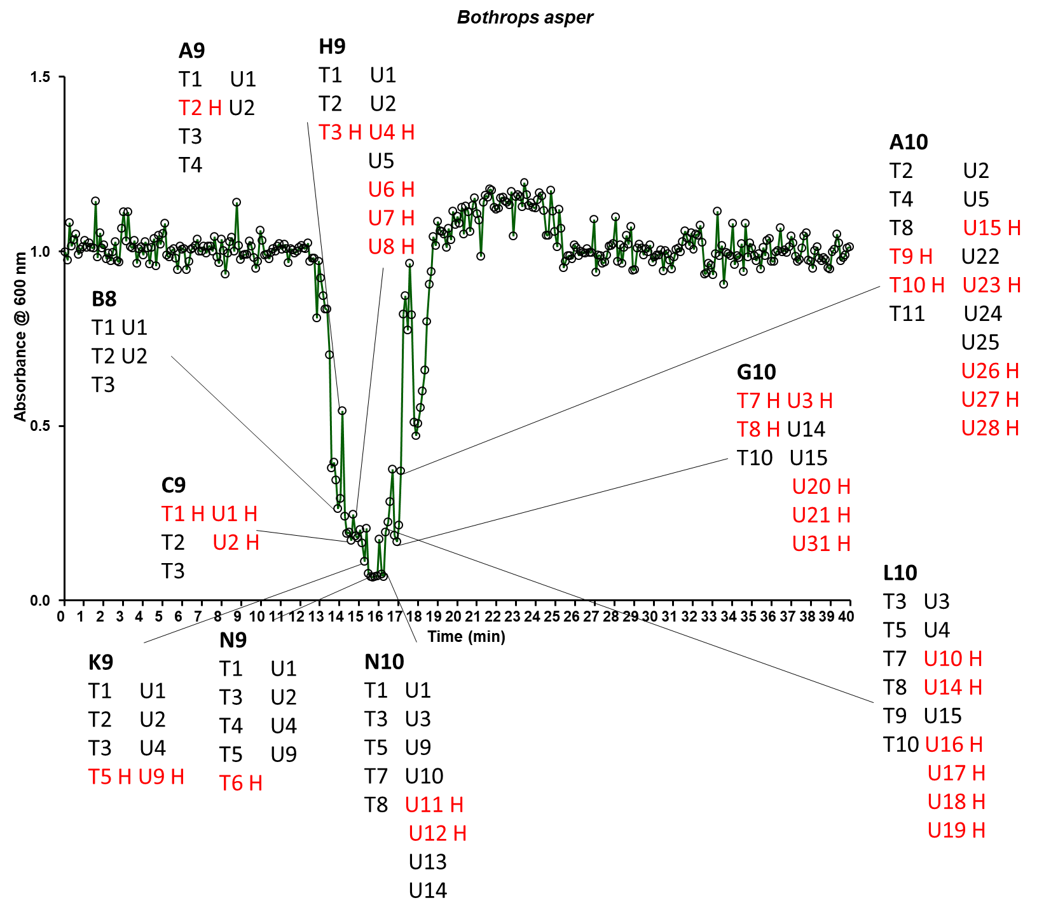

Supplement: S3 Fig — (ZIP) [file pntd.0007802.s003.zip › S1 Fig 3.5 Toxin IDs from the specific and non-specific databases.tif]

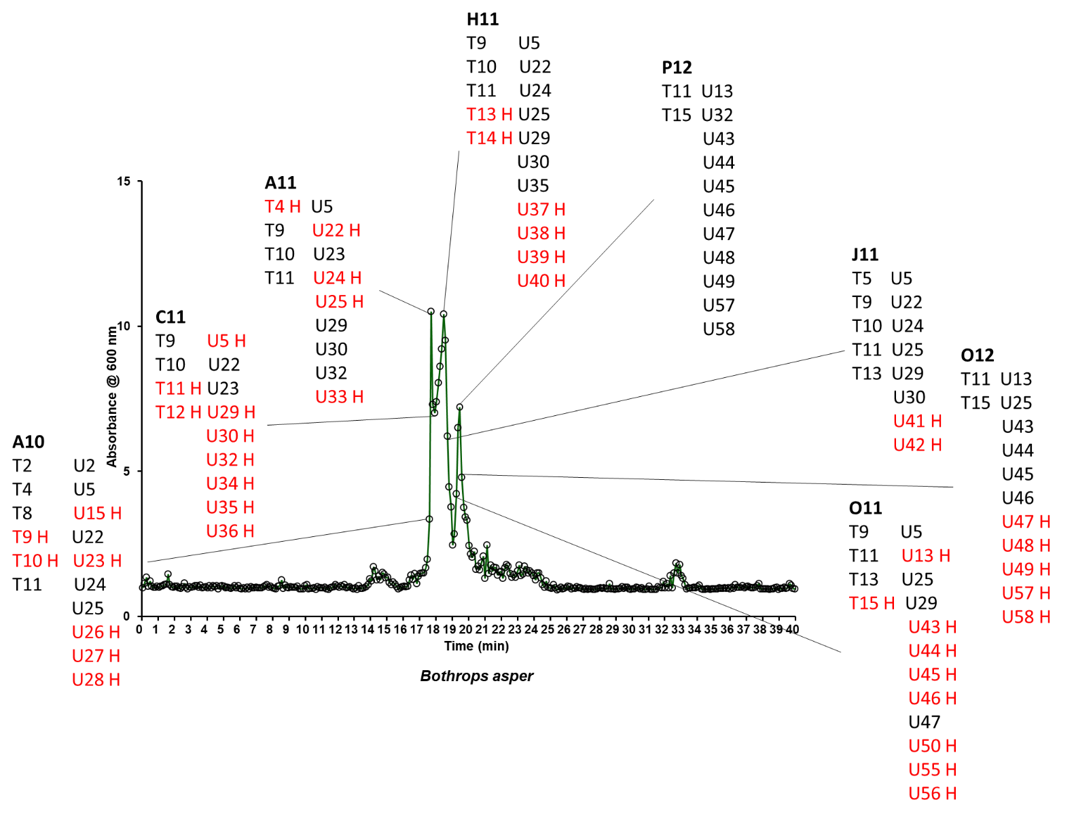

Supplement: S3 Fig — (ZIP) [file pntd.0007802.s003.zip › S1 Fig 3.6 Toxin IDs from the specific and non-specific databases.tif]

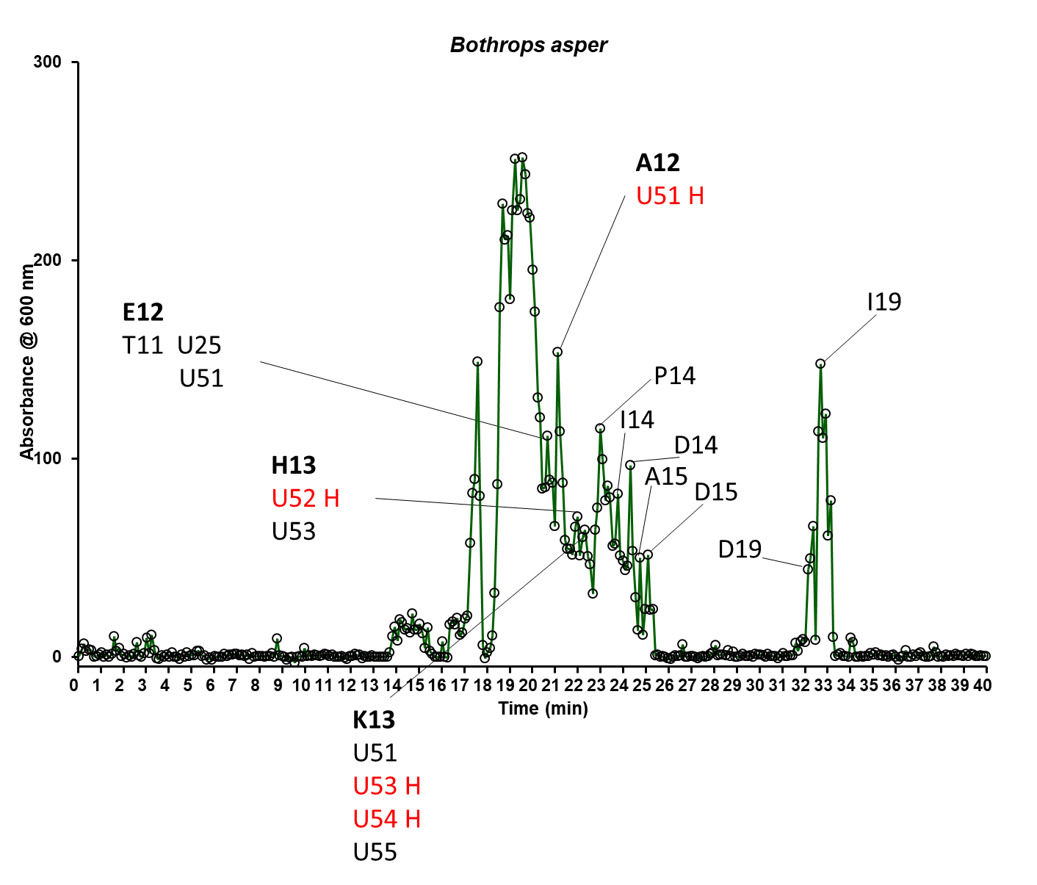

Supplement: S3 Fig — (ZIP) [file pntd.0007802.s003.zip › S1 Fig 3.7 Toxin IDs from the specific and non-specific databases.tif]

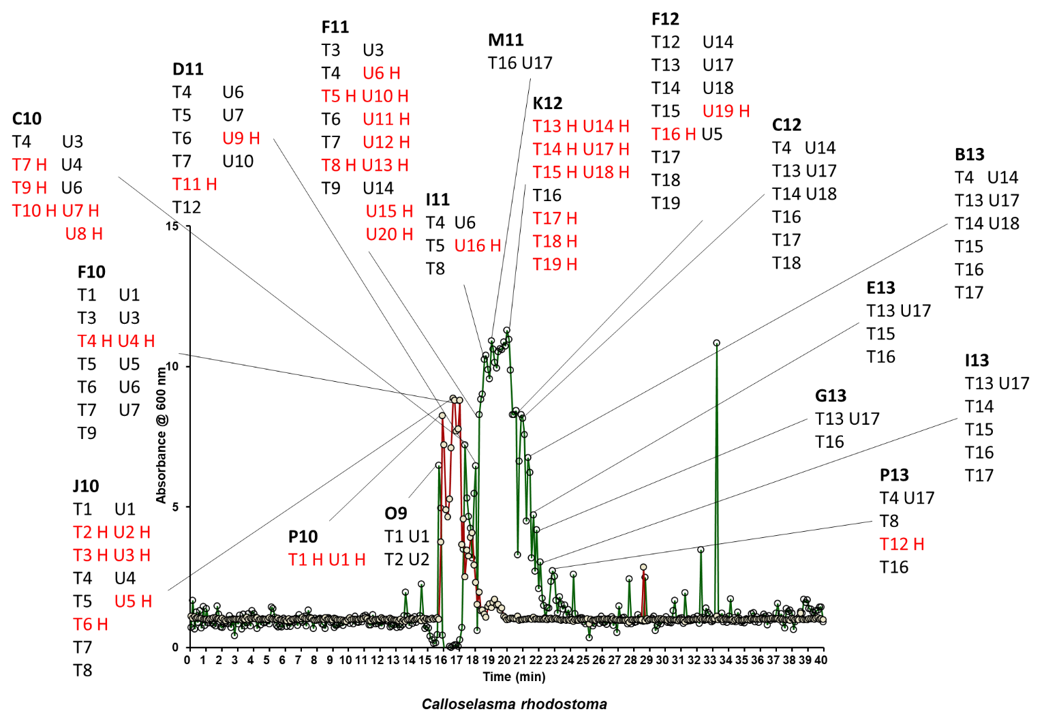

Supplement: S3 Fig — (ZIP) [file pntd.0007802.s003.zip › S1 Fig 3.8 Toxin IDs from the specific and non-specific databases.tif]

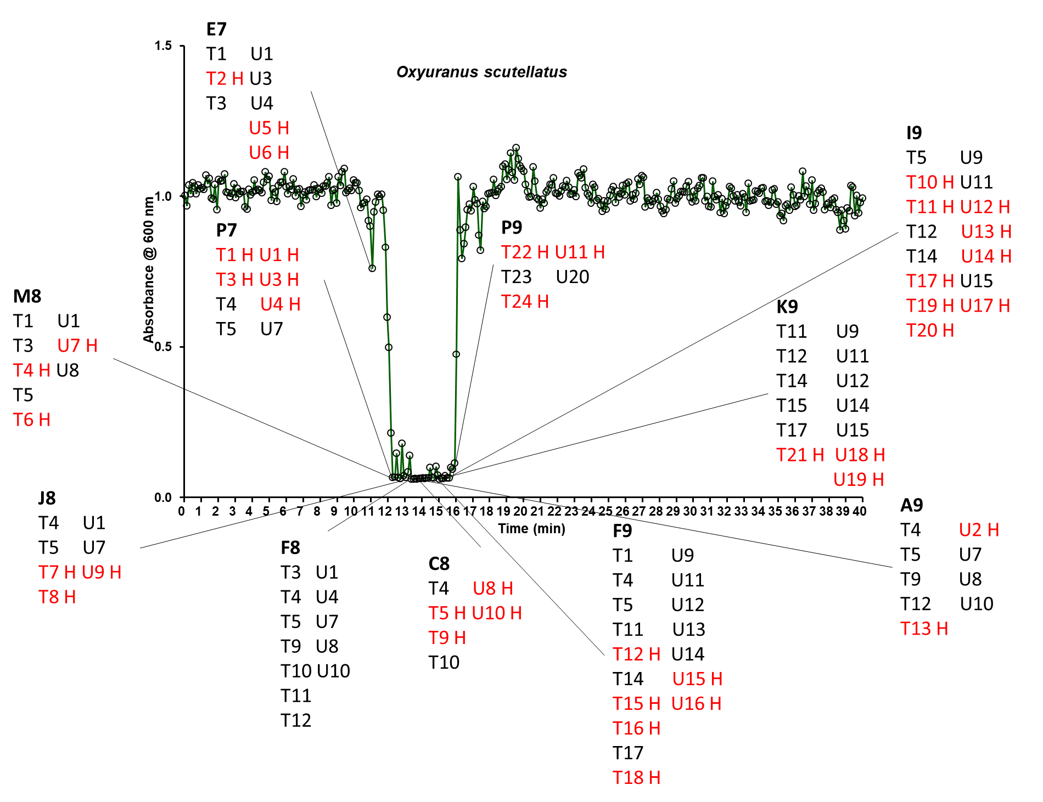

Supplement: S3 Fig — (ZIP) [file pntd.0007802.s003.zip › S1 Fig 3.9 Toxin IDs from the specific and non-specific databases.tif]
