# Supplementary material for: High throughput screening and identification of coagulopathic snake venom proteins and peptides using nanofractionation and proteomics approaches
Source: PLoS Negl Trop Dis. 2020 Apr 1;14(4):e0007802. doi: 10.1371/journal.pntd.0007802 (PMC7153897; doi:10.1371/journal.pntd.0007802)
Supplement: S1 Table — Table includes information on masses, retention times, well numbers of the nanofractionated toxins, sequence coverage, protein score, toxin class and coagulation activity. (DOCX) [file pntd.0007802.s004.docx]

| *Species* | Exact mass from MS data | Mascot results | Exact mass calculated from Mascot data | Retention time | Toxin ID | Well Numbers | Coverage % | Protein score | Toxin class | Activity |  |
| --- | --- | --- | --- | --- | --- | --- | --- | --- | --- | --- | --- |
| *Daboia russelli russelli* |  | Daboia_russelli_pulchella_1CL5_PLA2 | - | 13,6  15,0  17,0  18,3  18,8  19,1 | 1 | E8  I9  F10  G11  K11  N11 | 49  79  77  61  57  47 | 822  2092  2920  2037  1345  1036 | PLA2 | Anticoagulant |  |
|  |  | Daboia_russelli_siamensis_P0DL42_1_VEGF |  | 13,6  13,9  15.0 | 2 | E8  B8  I9 | 52  27  10 | 607  429  40 | VEGF | Anticoagulant |  |
|  |  | Daboia_russelli_1Q6V_chain_A_PLA2 |  | 13,9  15,0  19,3 | 3 | B8  I9  P11 | 14  58  11 | 84  1193  37 | PLA2 | Anticoagulant |  |
|  |  | Daboia_russelli_S28161_VNGF |  | 15,0 | 4 | I9 | 47 | 445 | VNGF | Anticoagulant |  |
|  |  | Daboia_russelli_CAA48457_1_PLA2 |  | 17,0 | 5 | F10 | 48 | 1052 | PLA2 | Anticoagulant |  |
|  |  | Daboia_russelli_siamensis_ADP88559_1_SVSP |  | 17,0  18,8 | 6 | F10  K11 | 6  11 | 79  120 | SVSP | Anticoagulant |  |
|  |  | Daboia_russelli_3SBK_SVSP |  | 17,0  19,1 | 7 | F10  N11 | 8  11 | 72  56 | SVSP | Procoagulant |  |
|  |  | Daboia_russelli_E5L0E4_1_SVSP |  | 18,3  18,8  19,3 | 8 | G11  K11  P11 | 17  11  11 | 805  433  153 | SVSP | Procoagulant |  |
|  |  | Daboia_russelli_VSP2_SVSP |  | 19,1  20,1 | 9 | N11  J12 | 86  86 | 87  19 | SVSP | Procoagulant |  |
|  |  | Daboia_russelli_AAW69870_1_CTL |  | 19,3  19,6  20,1 | 10 | P11  N12  J12 | 11  15  4 | 77  92  15 | CTL | Procoagulant |  |
|  |  | Daboia_russelli_AAW69869_1_CTL |  | 19,6 | 11 | N12 | 6 | 16 | CTL | Procoagulant |  |
|  |  |  |  |  |  |  |  |  |  |  |  |
| *Bothrops asper* |  |  |  |  |  |  |  |  |  |  |  |
|  |  | Bothrops_asper_AAF14241_PLA2 |  | 13,9  14,1  14,3  14,9  15,2  15,6  16,1 | 1 | B8  A9  C9  H9  K9  N9  N10 | 74  70  70  32  52  34  36 | 4967  10453  10653  1177  486  293  346 | PLA2 | Anticoagulant |  |
|  |  | Bothrops_asper_P24605_PLA1 |  | 13,9  14,1  14,3  14,9  17,5 | 2 | B8  A9  C9  H9  A10 | 72  72  72  25  18 | 2219  7578  5954  902  57 | PLA2 | Anticoagulant |  |
|  |  | Bothrops_asper_ABY55159_PLA2 |  | 13,9  14,1  14,3  14,9  15,2  15,6  16,1  16,3 | 3 | B8  A9  C9  H9  K9  N9  N10  L10 | 40  40  39  33  51  29  29  18 | 885  1832  1547  2180  606  375  201  134 | PLA2 | Anticoagulant |  |
|  |  | Bothrops_asper_1ND1_A_SVMP |  | 14,1  15,6  17,5  17,7 | 4 | A9  N9  A10  A11 | 2  5  32  38 | 23  35  850  1239 | SVMP | \| Procoagulant \|  \| \| --- \| --- \| |  |
|  |  | Bothrops_asper_1CLP_B_PLA2 |  | 15,2  15,6  16,1  16,3  18,6 | 5 | K9  N9  N10  L10  J11 | 48  27  37  35  6 | 355  184  253  153  23 | PLA2 | Anticoagulant |  |
|  |  | Bothrops_asper_AAB34336_PLA2 |  | 15,6 | 6 | N9 | 65 | 215 | PLA2 | Anticoagulant |  |
|  |  | Bothrops_asper_P20474_PLA2 |  | 16,1  16,3  16,9 | 7 | N10  L10  G10 | 47  63  67 | 448  1241  4784 | PLA2 | Anticoagulant |  |
|  |  | Bothrops_asper_5TFV_B_PLA2 |  | 16,1  16,3  16,9  17,5 | 8 | N10  L10  G10  A10 | 53  70  76  22 | 407  1083  4517  117 | PLA2 | Anticoagulant |  |
|  |  | Bothrops_asper_P86389_PLA2 |  | 16,3  17,5  17,7  17,9  18,4  18,6  19,2 | 9 | L10  A10  A11  C11  H11  J11  O11 | 14  71  73  28  11  11  5 | 195  2177  2129  132  63  51  24 | PLA2 | Anticoagulant |  |
|  |  | Bothrops_asper_ABB76280_SVSP |  | 16,3  16,9  17,5  17,7  17,9  18,4  18,6 | 10 | L10  G10  A10  A11  C11  H11  J11 | 8  3  23  22  19  5  3 | 408  53  467  43  29  190  17 | SVSP | Procoagulant |  |
|  |  | Bothrops_asper_ABB76281_SVMP_PII |  | 17,5  17,7  17,9  18,4  18,6  19,2  19,4  19,5  20,6 | 11 | A10  A11  C11  H11  J11  O11  P12  O12  E12 | 7  15  17  7  7  4  1  7  8 | 331  479  1339  377  228  78  38  334  322 | SVMP | Procoagulant |  |
|  |  | Bothrops_asper_2W15_A_SVMP |  | 17,9 | 12 | C11 | 62 | 2976 | SVMP | Procoagulant |  |
|  |  | Bothrops_asper_ABB76282_SVMP_PI |  | 18,4  18,6  19,2 | 13 | H11  J11  O11 | 16  14  9 | 977  524  72 | SVMP | Procoagulant |  |
|  |  | Bothrops_asper_P0DJC8_CTL |  | 18,4 | 14 | H11 | 17 | 70 | CTL | Procoagulant |  |
|  |  | Bothrops_asper_P84035_SVMP |  | 19,2  19,4  19,5 | 15 | O11  P12  O12 | 26  20  12 | 74  36  30 | SVMP | Procoagulant |  |
|  |  |  |  |  |  |  |  |  |  |  |  |
| *Calloselasma rhodostoma* |  |  |  |  |  |  |  |  |  |  |  |
|  |  | Calloselasma_rhodostoma_VGT1642_VGT4432_R_3.4943_L_674_PLA2 |  | 15,7  15,9  16,5  17,0  17,3 | 1 | O9  P10  J10  F10  C10 | 77  77  44  40  5 | 2994  4198  877  272  29 | PLA2 | Anticoagulant |  |
|  |  | Calloselasma_rhodostoma_VGT3678_R_1.3161_L_405_SVMP |  | 15,7  16,5 | 2 | O9  J10 | 16  16 | 113  148 | SVMP | Procoagulant |  |
|  |  | Calloselasma_rhodostoma_VGT0932_R_0.1931_L_908_Serine_Protease |  | 16,5  17,0  18,2 | 3 | J10  F10  F11 | 32  32  9 | 1676  674  37 | SVSP | Procoagulant |  |
|  |  | Calloselasma_rhodostoma_VGT1895_R_0.6337_L_623_Ctype_Lectin |  | 16,5  17,0  17,3  18,0  18,2  18,5  20,8  21,3  21,8 | 4 | J10  F10  C10  D11  F11  I11  C12  B13  P13 | 41  65  44  10  6  4  4  4  19 | 1056  4348  432  28  16  26  15  24  38 | CTL | Procoagulant |  |
|  |  | Calloselasma_rhodostoma_VGT0086_R_0.0309_L_2109_Serine_Protease |  | 16,5  17,0  18,0  18,2  18,5 | 5 | J10  F10  D11  F11  I11 | 5  5  5  10  5 | 213  119  191  222  48 | SVSP | Procoagulant |  |
|  |  | Calloselasma_rhodostoma_VGT4464_R_1.4675_L_300_Ctype_Lectin |  | 16,5  17,0  18,0  18,2 | 6 | J10  F10  D11  F11 | 20  16  16  9 | 193  84  24  29 | CTL | Procoagulant |  |
|  |  | Calloselasma_rhodostoma_VGT1329_R_0.1644_L_748_PLA2 |  | 16,5  17,0  17,3  18,0  18,2 | 7 | J10  F10  C10  D11  F11 | 6  12  60  23  6 | 82  224  1491  65  29 | PLA2 | Procoagulant |  |
|  |  | Calloselasma_rhodostoma_VGT0432_R_0.0247_L_1286_Serine_Protease |  | 16,5  18,2  18,5  22,8 | 8 | J10  F11  I11  P13 | 11  20  10  8 | 23  488  47  19 | SVSP | Procoagulant |  |
|  |  | Calloselasma_rhodostoma_VGT1180_R_0.3748_L_800_Serine_Protease |  | 17,0  17,3  18,2 | 9 | F10  C10  F11 | 40  63  7 | 668  5193  149 | SVSP | Procoagulant |  |
|  |  | Calloselasma_rhodostoma_VGT0715_R_0.0078_L_1021_Serine_Protease |  | 17,3 | 10 | C10 | 4 | 17 | SVSP | Procoagulant |  |
|  |  | Calloselasma_rhodostoma_VGT0469_R_0.0525_L_1241_Serine_Protease |  | 18,0 | 11 | D11 | 8 | 42 | SVSP | Procoagulant |  |
|  |  | Calloselasma_rhodostoma_VGT2768_R_0.0442_L_496_Ctype_Lectin |  | 18,0  18,2  20,5  22,8 | 12 | D11  F11  F12  P13 | 4  4  4  9 | 16  23  20  23 | CTL | Procoagulant |  |
|  |  | Calloselasma_rhodostoma_VGT1518_R_0.1873_L_700_SVMP |  | 18,2  20,0  20,5  20,8  21,3  21,6  21,8  22,1 | 13 | F11  K12  F12  C12  B13  E13  G13  I13 | 7  7  12  12  12  12  7  12 | 212  873  461  177  163  44  76  131 | SVMP | Procoagulant |  |
|  |  | Calloselasma_rhodostoma_VGT4398_VGT0985_VGT2596_R_10.6741_L_313_SVMP |  | 18,2  20,0  20,5  20,8  21,3  22,1 | 14 | F11  K12  F12  C12  B13  I13 | 4  9  9  9  9  9 | 54  646  234  131  147  136 | SVMP | Procoagulant |  |
|  |  | Calloselasma_rhodostoma_VGT4466_R_6.2285_L_300_SVMP |  | 18,2  18,5  20,0  20,5  21,3  21,6  22,1 | 15 | F11  I11  K12  F12  B13  E13  I13 | 17  11  51  50  36  11  17 | 31  16  1031  427  91  16  35 | SVMP | Procoagulant |  |
|  |  | Calloselasma_rhodostoma_VGT0003_R_0.276_L_4023_Lamino_Oxidase |  | 19,0  20,0  20,5  20,8  21,3  21,6  21,8  22,1  22,8 | 16 | M11  K12  F12  C12  B13  E13  G13  I13  P13 | 6  19  19  14  14  3  6  6  3 | 252  338  461  139  275  58  120  168  41 | LAAO | Procoagulant |  |
|  |  | Calloselasma_rhodostoma_VGT4456_VGT1757_VGT1858_R_0.4549_L_300_Lamino_Oxidase |  | 20,0  20,5  20,8  21,3  22,1 | 17 | K12  F12  C12  B13  I13 | 19  6  10  11  6 | 334  171  133  54  46 | LAAO | Procoagulant |  |
|  |  | Calloselasma_rhodostoma_VGT3173_R_1.1662_L_455_SVMP |  | 20,0  20,5  20,8 | 18 | K12  F12  C12 | 33  26  14 | 327  212  73 | SVMP | Procoagulant |  |
|  |  | Calloselasma_rhodostoma_VGT0031_R_0.2018_L_2650_SVMP |  | 20,0  20,5 | 19 | K12  F12 | 12  13 | 217  39 | SVMP | Procoagulant |  |
|  |  |  |  |  |  |  |  |  |  |  |  |
|  |  |  |  |  |  |  |  |  |  |  |  |
| *Oxyuranus scutelatus* |  |  |  |  |  |  |  |  |  |  |  |
|  |  | sp\|A8HDK9.1\|3L21_OXYSC |  | 11,0  12,2  12,7  14,7 | 1 | E7  P7  M8  F9 | 55  55  10  10 | 560  972  58  18 | Neurotoxin | Anticoagulant |  |
|  |  | sp\|P0CB06.1\|3S12_OXYSC |  | 11,0 | 2 | E7 | 22 | 235 | Neurotoxin | Anticoagulant |  |
|  |  | sp\|B7S4N9.1\|VKT_OXYSC |  | 11,0  12,2  12,7  13,5  16,5 | 3 | E7  P7  M8  F8  J10 | 21  84  59  18  10 | 54  2029  939  78  19 | Serine protease inhibitor | Anticoagulant |  |
|  |  | AAY47067.1 |  | 12,2  12,7  13,0  13,5  13,8  14,1  14,7  16,9 | 4 | P7  M8  J8  F8  C8  A9  F9  G10 | 38  38  43  43  30  42  20  10 | 605  1396  647  1212  367  353  537  156 | alpha taipoxin-2 precursor | Anticoagulant |  |
|  |  | sp\|P00614.1\|PA2TA_OXYSC |  | 12,2  12,7  13,0  13,5  13,8  14,1  14,7  15,0  17,1 | 5 | P7  M8  J8  F8  P8  A9  F9  I9  E10 | 17  18  39  56  56  47  33  27  21 | 157  475  497  1427  1874  693  353  314  151 | PLA2 | Anticoagulant |  |
|  |  | AAZ22652.1 (*Notechis scutatus)* |  | 12,7 | 6 | M8 | 9 | 21 | PLA2 precursor | Anticoagulant |  |
|  |  | pdb\|3VC0\|A |  | 13,0 | 7 | J8 | 87 | 1935 | Chain A, Crystal Structure Of Taipoxin Beta Subunit Isoform 1 | Anticoagulant |  |
|  |  | ACC77765.1 |  | 13,0 | 8 | J8 | 40 | 166 | taicatoxin serine protease inhibitor precursor | Anticoagulant |  |
|  |  | AAZ22636.1 |  | 13,5  13,8  14,1 | 9 | F8  C8  A9 | 53  44  46 | 874  955  557 | PLA-6 precursor | Anticoagulant |  |
|  |  | AAZ22640.1 |  | 13,5  13,8  15,0 | 10 | F8  C8  I9 | 31  31  55 | 353  565  1438 | PLA-2 precursor [*Oxyuranus microlepidotus*] | Anticoagulant |  |
|  |  | AAY47066.1 |  | 13,5  14,7  15,0  15,2  16,2 | 11 | F8  F9  I9  K9  M10 | 25  70  71  71  33 | 256  878  1695  1672  254 | beta taipoxin precursor | Anticoagulant |  |
|  |  | AAY47072.1 |  | 13,5  14,1  14,7  15,0  15,2  16,2 | 12 | F8  A9  F9  I9  K9  M10 | 12  12  20  18  20  21 | 56  68  164  96  146  45 | natriuretic peptide | Anticoagulant |  |
|  |  | sp\|B5KL29.1\|VKT3_OXYSC |  | 14,1 | 13 | A9 | 65 | 578 | Serine protease inhibitor | Anticoagulant |  |
|  |  | AAB33759.1 |  | 14,7  15,0  15,2  16,2  16,5  17,1 | 14 | F9  I9  K9  M10  J10  E10 | 77  74  77  78  74  26 | 2614  1601  4773  5175  1339  128 | OS1=secretory phospholipase A2 (PLA2) | Anticoagulant |  |
|  |  | pdb\|3VBZ\|A |  | 14,7  15,2 | 15 | F9  K9 | 64  65 | 1861  693 | Chain A, Crystal Structure Of Taipoxin Beta Subunit Isoform 2 | Anticoagulant |  |
|  |  | AAZ22638.1 |  | 14,7 | 16 | F9 | 29 | 524 | beta paradoxin-like precursor | Anticoagulant |  |
|  |  | sp\|Q3I5F4.1\|NGFV_OXYSC |  | 14,7  15,0  15,2  16,2  16,9  17,1 | 17 | F9  I9  K9  M10  G10  E10 | 19  29  19  4  6  4 | 555  560  468  30  101  56 | NGFV | Anticoagulant |  |
|  |  | sp\|Q58L91.1\|FA5V_OXYSU |  | 14,7 | 18 | F9 | 1 | 22 | Venom prothrombin activator oscutarin-C non-catalytic subunit | Anticoagulant |  |
|  |  | sp\|P0CG57.1\|PA2TC_OXYSC |  | 15,0 | 19 | I9 | 65 | 4355 | PLA2 | Anticoagulant |  |
|  |  | AAZ22634.1 |  | 15,0 | 20 | I9 | 63 | 3732 | PLA-4 precursor | Anticoagulant |  |
|  |  | sp\|A8HDK2.1\|3SX3_OXYSC |  | 15,2  16,2  16,9 | 21 | K9  M10  G10 | 18  12  12 | 35  21  38 | Neurotoxin | Anticoagulant |  |
|  |  | sp\|Q4VRI5.1\|PA21_OXYSC |  | 15,8  16,9 | 22 | P9  G10 | 60  63 | 2701  511 | PLA2 | Anticoagulant |  |
|  |  | sp\|Q3SAX8.1\|VNPD_OXYSC |  | 15,8  16,2  16,5  16,9  17,1 | 23 | P9  M10  J10  G10  E10 | 53  53  80  80  24 | 148  145  295  578  128 | Natriuretic peptide | Anticoagulant |  |
|  |  | sp\|D2YVK1.1\|LECG_HOPST |  | 15,8 | 24 | P9 | 3 | 21 | C-type lectin galactose-binding isoform | Anticoagulant |  |
|  |  | AAZ22635.1 |  | 16,2 | 25 | M10 | 20 | 141 | PLA-5 precursor | Anticoagulant |  |
|  |  | AAY47069.1 |  | 16,5 | 26 | J10 | 76 | 1266 | OS5 precursor | Procoagulant |  |
|  |  | sp\|Q3SB05.1\|CRVP_PSETE |  | 16,9 | 27 | G10 | 3 | 65 | Cysteine-rich venom protein pseudechetoxin-like | Procoagulant |  |
|  |  | sp\|Q4JHE1.1\|OXLA_PSEAU |  | 19,3 | 28 | P11 | 3 | 208 | Pseudechis australis LAOO | Procoagulant |  |
|  |  |  |  |  |  |  |  |  |  |  |  |
| *Echis ocellatus* |  |  |  |  |  |  |  |  |  |  |  |
|  |  | Echis_ocellatus_03A04_EOC00167_LAAO |  | 12,8  19,4 | 1 | L8  P12 | 4  11 | 66  39 | LAAO | Procoagulant |  |
|  |  | Echis_ocellatus_04C11_EOC00015_PLA2 |  | 13,9 | 2 | B8 | 72 | 3117 | PLA2 | Anticoagulant |  |
|  |  | Echis_ocellatus_EOC00063_83523627_SVMP |  | 13,9  20,1  20,5 | 3 | B8  J12  F12 | 9  29  19 | 705  1673  862 | SVMP | Procoagulant |  |
|  |  | Echis_ocellatus_EOC00001_83523625_SVMP |  | 13,9 | 4 | B8 | 8 | 310 | SVMP | Anticoagulant |  |
|  |  | Echis_ocellatus_EOC00022_83523635_SVMP |  | 13,9  19,7  20,1  20,5 | 5 | B8  M12  J12  F12 | 3  38  35  1 | 46  4511  3208  67 | SVMP | Procoagulant |  |
|  |  | Echis_ocellatus_03E06_EOC00140_PLA2 |  | 16,1 | 6 | N10 | 26 | 173 | PLA2 | Anticoagulant |  |
|  |  | Echis_ocellatus_01A10_EOC00265_CTL |  | 19,7  20,1 | 7 | M12  J12 | 48  48 | 424  411 | CTL | Procoagulant |  |
|  |  | Echis_ocellatus_02C06_EOC00124_CTL |  | 19,7  20,1  20,5 | 8 | M12  J12  F12 | 17  17  8 | 38  19  54 | CTL | Procoagulant |  |
|  |  | Echis_ocellatus_04D06_EOC00087_CTL |  | 19,7  20,1  20,5 | 9 | M12  J12  F12 | 5  5  9 | 30  23  75 | CTL | Procoagulant |  |
|  |  | Echis_ocellatus_EOC00095_83523631_SVMP |  | 20,1  20,5 | 10 | J12  F12 | 9  8 | 558  275 | SVMP | Procoagulant |  |
|  |  | Echis_ocellatus_EOC00013_83523633_SVMP |  | 20,1  20,5 | 11 | J12  F12 | 15  8 | 312  160 | SVMP | Procoagulant |  |
|  |  | Echis_ocellatus_04F08_EOC00073_SVMP |  | 20,1  20,5 | 12 | J12  F12 | 22  11 | 272  52 | SVMP | Procoagulant |  |
|  |  | Echis_ocellatus_EOC00089_83523641_SVMP |  | 20,1  20,5  21,1 | 13 | J12  F12  A12 | 4  3  26 | 161  36  966 | SVMP | Procoagulant |  |
|  |  | Echis_ocellatus_EOC00008_SVMP |  | 20,1 | 14 | J12 | 5 | 66 | SVMP | Procoagulant |  |
|  |  | Echis_ocellatus_EOC00404_83523645_SVMP |  | 20,5  21,1 | 15 | F12  A12 | 21  17 | 663  487 | SVMP | Procoagulant |  |
|  |  |  |  |  |  |  |  |  |  |  |  |
| *Bothrops jararaca* |  |  |  |  |  |  |  |  |  |  |  |
|  |  | BJAR_SVMP_03ed_SVMP |  | 13,9  17,3  21,8  22,2  23,9  31,9  32,6  33,0 | 1 | B8  C10  G13  J13  G14  A19  I19  L19 | 12  1  15  40  10  13  5  4 | 1051  26  102  7395  507  442  244  266 | SVMP | Procoagulant |  |
|  |  | BJAR_SVMP_20_2_SVMP |  | 13,9 | 2 | B8 | 2 | 65 | SVMP | Anticoagulant |  |
|  |  | BJAR_iso1_PLA2 |  | 13,9  14,9  15,6 | 3 | B8  H9  N9 | 15  16  29 | 60  75  287 | PLA2 | Anticoagulant |  |
|  |  | BJAR_SVMP_19ed_SVMP |  | 14,9 | 4 | H9 | 2 | 121 | SVMP | Anticoagulant |  |
|  |  | BJARGLA00259_NGF |  | 14,9  15,6 | 5 | H9  N9 | 7  10 | 80  114 | NGF | Anticoagulant |  |
|  |  | BJAR_iso6_PLA2 |  | 14,9 | 6 | H9 | 6 | 46 | PLA2 | Anticoagulant |  |
|  |  | BJARALL26960_CRISP |  | 16,4 | 7 | K10 | 57 | 1761 | Cysteine rich venom protein | Anticoagulant |  |
|  |  | BJAR_SVSPisotig00016_SVSP |  | 16,4  17,0 | 8 | K10  F10 | 38  34 | 1043  414 | SVSP | Procoagulant |  |
|  |  | BJAR_SVSPisotig00021_SVSP |  | 16,4  17,0  17,4  17,5  17,8  18,0 | 9 | K10  F10  B10  A10  B11  D11 | 27  17  10  31  47  24 | 570  173  185  137  427  302 | SVSP | Procoagulant |  |
|  |  | BJAR_SVSPisotig00008_SVSP |  | 17,0  17,3  17,4  17,5  17,8 | 10 | F10  C10  B10  A10  B11 | 51  22  29  15  14 | 1551  347  423  124  162 | SVSP | Procoagulant |  |
|  |  | BJAR_CTL14_CTL |  | 17,0  17,3  17,4  17,5  17,8  18,0 | 11 | F10  C10  B10  A10  B11  D11 | 63  58  53  30  22  7 | 1174  776  704  134  35  20 | CTL | Procoagulant |  |
|  |  | BJARALL26960_CRISP |  | 17,0  17,3 | 12 | F10  C10 | 15  4 | 143  27 | Cystein rich venom protein | Procoagulant |  |
|  |  | BJAR_iso3_PLA2 |  | 17,3  17,4  17,5  17,8  18,0  18,5  19,0 | 13 | C10  B10  A10  B11  D11  I11  M11 | 47  47  35  5  15  27  26 | 518  956  179  31  50  71  56 | PLA2 | Procoagulant |  |
|  |  | BJAR_SVSPisotig00029_SVSP |  | 17,3  17,4  17,5  17,8  18,0  18,5 | 14 | C10  B10  A10  B11  D11  I11 | 40  47  23  13  13  5 | 451  573  127  151  108  48 | SVSP | Procoagulant |  |
|  |  | BJAR_SVSPisotig00017_SVSP |  | 17,3  17,8  18,0 | 15 | C10  B11  D11 | 16  32  37 | 340  265  367 | SVSP | Procoagulant |  |
|  |  | BJAR_SVSPisotig00001_SVSP |  | 17,3  17,4  17,8  18,0  18,5  19,2  20,0  20,3  20,8  21,8  22,2  23,9  31,8 | 16 | C10  B10  B11  D11  I11  O11  K12  H12  C12  G13  J13  G14  A19 | 11  11  31  38  42  7  9  31  17  18  8  27  34 | 184  84  195  908  295  38  106  156  110  86  112  172  107 | SVSP | Procoagulant |  |
|  |  | BJAR_SVSPisotig00014_SVSP |  | 17,3  17,4  17,5  17,8 | 17 | C10  B10  A10  B11 | 16  43  41  29 | 179  843  815  178 | SVSP | Procoagulant |  |
|  |  | BJAR_SVSPisotig00012_SVSP |  | 17,3  17,4  17,5  17,8  21,8 | 18 | C10  B10  A10  B11  G13 | 7  13  27  14  10 | 87  194  132  99  27 | SVSP | Procoagulant |  |
|  |  | BJAR_SVSPisotig00022ed_SVSP |  | 17,4  17,5  17,8  18,0  18,5 | 19 | B10  A10  B11  D11  I11 | 25  45  53  37  10 | 185  715  1851  973  141 | SVSP | Procoagulant |  |
|  |  | BJAR_SVSPisotig00002_SVSP |  | 17,8  18,0 | 20 | B11  D11 | 21  18 | 174  197 | SVSP | Procoagulant |  |
|  |  | BJAR_SVSPisotig00032_SVSP |  | 17,8  18,0  18,5 | 21 | B11  D11  I11 | 22  33  19 | 109  196  40 | SVSP | Procoagulant |  |
|  |  | BJAR_SVSPisotig00003ed_SVSP |  | 18,0  18,5 | 22 | D11  I11 | 29  58 | 544  962 | SVSP | Procoagulant |  |
|  |  | BJAR_SVMP_04edRev_SVMP |  | 19,0  19,2 | 23 | M11  O11 | 10  12 | 135  99 | SVMP | Procoagulant |  |
|  |  | BJAR_SVMP_22edRev_SVMP |  | 19,0  20,0  20,3 | 24 | M11  K12  H12 | 7  11  7 | 128  272  120 | SVMP | Procoagulant |  |
|  |  | BJAR_SVMP_48edRev_SVMP |  | 19,0  19,2  20,0  20,3  31,8 | 25 | M11  O11  K12  H12  A19 | 5  3  3  3  3 | 25  45  62  25  20 | SVMP | Procoagulant |  |
|  |  | BJAR_LAAO_1 |  | 19,0  19,2  20,0 | 26 | M11  O11  K12 | 10  17  6 | 108  158  45 | LAAO | Procoagulant |  |
|  |  | BJAR_CTL21a_CTL |  | 19,0  19,2  20,0  20,3  20,8  21,8  22,2  23,9  31,8 | 27 | M11  O11  K12  H12  C12  G13  J13  G14  A19 | 10  19  19  19  19  26  19  26  10 | 98  72  101  134  104  130  149  178  82 | CTL | Procoagulant |  |
|  |  | BJAR_CTLX2_CTL |  | 19,0  19,2  20,0  20,3  20,8  31,8 | 28 | M11  O11  K12  H12  C12  A19 | 12  9  9  9  9  17 | 76  54  38  26  42  90 | CTL | Procoagulant |  |
|  |  | BJAR_CTL07_CTL |  | 19,0  19,2  20,0  20,3  20,8  21,8  22,2  23,9  31,8 | 29 | M11  O11  K12  H12  C12  G13  J13  G14  A19 | 17  22  13  22  22  17  17  24  21 | 58  107  68  66  112  93  117  83  78 | CTL | Procoagulant |  |
|  |  | BJAR_SVMP_17ed_SVMP |  | 19,2  31,8 | 30 | O11  A19 | 16  2 | 371  33 | SVMP | Procoagulant |  |
|  |  | BJAR_SVMP_21ed_SVMP |  | 19,2  19,4  20,0 | 31 | O11  P12  K12 | 23  9  15 | 250  59  88 | SVMP | Procoagulant |  |
|  |  | BJAR_SVMP_19ed_SVMP |  | 19,2  19,4 | 32 | O11  P12 | 25  11 | 234  55 | SVMP | Procoagulant |  |
|  |  | BJAR_CTL09_CTL |  | 19,2  20,3  21,8 | 33 | O11  H12  G13 | 14  14  14 | 40  16  25 | CTL | Procoagulant |  |
|  |  | BJAR_CTLX1_CTL |  | 20,3  23,9 | 34 | H12  G14 | 18  20 | 159  41 | CTL | Procoagulant |  |
|  |  | BJARALL26935ed_CTL |  | 20,3 | 35 | H12 | 8 | 28 | CTL | Procoagulant |  |
|  |  | BJAR_SVMP_18edRev_SVMP |  | 20,8 | 36 | C12 | 5 | 49 | SVMP | Procoagulant |  |
|  |  | BJAR_SVMP_07_1Rev_SVMP |  | 21,8  23,9 | 37 | G13  G14 | 3  2 | 71  40 | SVMP | Procoagulant |  |
|  |  | BJAR_SVMP_24edRev_SVMP |  | 21,8 | 38 | G13 | 5 | 56 | SVMP | Procoagulant |  |
|  |  | BJAR_SVMP_02ed_SVMP |  | 21,8 | 39 | G13 | 2 | 23 | SVMP | Procoagulant |  |
|  |  | BJAR_SVMP_26_SVMP |  | 23,9 | 40 | G14 | 3 | 37 | SVMP | Procoagulant |  |
|  |  | BJAR_iso5_PLA2 |  | 23,9 | 41 | G14 | 7 | 20 | PLA2 | Procoagulant |  |
|  |  | BJARALL24975_cystatin |  | 23,9 | 42 | G14 | 5 | 18 | Cystatin | Procoagulant |  |
|  |  |  |  |  |  |  |  |  |  |  |  |
|  |  |  |  |  |  |  |  |  |  |  |  |
|  |  |  |  |  |  |  |  |  |  |  |  |
| *Dispholidus typus* |  |  |  |  |  |  |  |  |  |  |  |
|  |  | Dispholidus_typus_BMSLG-T1154_R_2.7403_L_685_PLA2_group_IIE |  | 14,0 | 1 | A8 | 32 | 639 | PLA2 | Anticoagulant |  |
|  |  | Dispholidus_typus_BMSLGT0050_R_3.3713_L_2386_SVMP |  | 14,0  17,9  18,6  19,0  19,9  20,4  21,4 | 2 | A8  C11  J11  M11  L12  G12  C13 | 4  1  8  3  5  17  13 | 164  21  84  47  43  777  429 | SVMP | Procoagulant |  |
|  |  | Dispholidus_typus_BMSLGT0692_R_0.2091_L_868_SVMP_partial |  | 14,0  20,4 | 3 | A8  G12 | 14  14 | 15  175 | SVMP | Procoagulant |  |
|  |  | Dispholidus_typus_BMSLG-T0114_T3034_merged_R_1.9585_L_1841 |  | 17,9  18,6 | 4 | C11  J11 | 64  14 | 2813  257 | Cysteine rich venom protein | Procoagulant |  |
|  |  | Dispholidus_typus_BMSLGT0144_R_0.1951_L_1697_SVMP_partial |  | 17,9 | 5 | C11 | 1 | 23 | SVMP | Procoagulant |  |
|  |  | Dispholidus_typus_BMSLGT0510_R_0.5491_L_1000_SVMP_partial |  | 18,6  19,9 | 6 | J11  L12 | 9  13 | 299  177 | SVMP | Procoagulant |  |
|  |  | Dispholidus_typus_Consensus2_R_5.0541_SVMP |  | 18,6  19,0  19,3  19,9 | 7 | J11  M11  P11  L12 | 17  15  18  15 | 268  121  375  235 | SVMP | Procoagulant |  |
|  |  | Dispholidus_typus_BMSLGT0144_R_0.1951_L_1697_SVMP_partial |  | 18,6  19,0  19,9 | 8 | J11  M11  L12 | 2  6  5 | 164  148  126 | SVMP | Procoagulant |  |
|  |  | Dispholidus_typus_Consensus1_21.0694_SVMP |  | 18,6  19,0  19,9  20,4 | 9 | J11  M11  L12  G12 | 3  10  7  6 | 119  202  247  121 | SVMP | Procoagulant |  |
|  |  | Dispholidus_typus_BMSLGT3502_R_1.4368_L_350_SVMP_partial |  | 18,6  19,0 | 10 | J11  M11 | 30  19 | 113  101 | SVMP | Procoagulant |  |
|  |  | Dispholidus_typus_BMSLGT0991_R_1.3553_L_728_SVMP_partial |  | 19,0  19,3  19,9  22,2 | 11 | M11  P11  L12  J13 | 17  20  17  3 | 207  375  344  23 | SVMP | Procoagulant |  |
|  |  | Dispholidus_typus_BMSLGT0056_R_1.6322_L_2317_SVMP |  | 19,0  19,3  19,9 | 12 | M11  P11  L12 | 7  15  7 | 113  1225  295 | SVMP | Procoagulant |  |
|  |  | Dispholidus_typus_BMSLGT0014_R_1.6061_L_3335_SVMP |  | 19,3  20,4 | 13 | P11  G12 | 12  9 | 514  117 | SVMP | Procoagulant |  |
|  |  | Dispholidus_typus_BMSLG-T0197_R_1.9105_L_1524_serine_protease |  | 19,9  20,4  21,4  22,2  23,3  23,7 | 14 | L12  G12  C13  J13  M14  I14 | 32  27  17  9  5  5 | 1250  513  99  33  44  138 | SVSP | Procoagulant |  |
|  |  | Dispholidus_typus_BMSLGT2532_R_1.5939_L_450_SVMP_partial |  | 19,9 | 15 | L12 | 24 | 300 | SVMP | Procoagulant |  |
|  |  | Dispholidus_typus_BMSLGT0060_R_0.5139_L_2297_SVMP |  | 19,9  20,4 | 16 | L12  G12 | 5  5 | 79  31 | SVMP | Procoagulant |  |
|  |  | Dispholidus_typus_Consensus3_R_0.9425_SVMP |  | 19,9  21,4 | 17 | L12  C13 | 20  9 | 64  53 | SVMP | Procoagulant |  |
|  |  | Dispholidus_typus_BMSLGT3035_R_1.9219_L_400_SVMP_partial |  | 20,4  21,4 | 18 | G12  C13 | 25  17 | 196  67 | SVMP | Procoagulant |  |
|  |  | Dispholidus_typus_BMSLGT0509_R_0.3771_L_1000_SVMP_partial |  | 20,4  21,4 | 19 | G12  C13 | 25  21 | 158  21 | SVMP | Procoagulant |  |
|  |  | Dispholidus_typus_BMSLGT2073_R_0.3911_L_500_SVMP_partial |  | 20,4 | 20 | G12 | 23 | 103 | SVMP | Procoagulant |  |
|  |  | Dispholidus_typus_BMSLGT0569_R_0.3619_L_950_SVMP_partial |  | 21,4 | 21 | C13 | 6 | 126 | SVMP | Procoagulant |  |
|  |  | Dispholidus_typus_BMSLGT0692_R_0.2091_L_868_SVMP_partial |  | 21,4 | 22 | C13 | 14 | 125 | SVMP | Procoagulant |  |
|  |  |  |  |  |  |  |  |  |  |  |  |
|  |  |  |  |  |  |  |  |  |  |  |  |
|  |  |  |  |  |  |  |  |  |  |  |  |
|  |  |  |  |  |  |  |  |  |  |  |  |
|  |  |  |  |  |  |  |  |  |  |  |  |
|  |  |  |  |  |  |  |  |  |  |  |  |
|  |  |  |  |  |  |  |  |  |  |  |  |
|  |  |  |  |  |  |  |  |  |  |  |  |
|  |  |  |  |  |  |  |  |  |  |  |  |

**SI Species specific databases table.** All Mascot hits found with the species specific databases in the area where pro- and anticoagulant activity was observed. Table includes information on masses, retention times, well numbers of the nanofractionated toxins, sequence coverage, protein score, toxin class and coagulation activity.
