# Supplementary material for: High throughput screening and identification of coagulopathic snake venom proteins and peptides using nanofractionation and proteomics approaches
Source: PLoS Negl Trop Dis. 2020 Apr 1;14(4):e0007802. doi: 10.1371/journal.pntd.0007802 (PMC7153897; doi:10.1371/journal.pntd.0007802)
Supplement: S2 Table — Table includes information on masses, retention times, well numbers of the nanofractionated toxins, sequence coverage, protein score, toxin class and coagulation activity. (DOCX) [file pntd.0007802.s005.docx]

| *Species* | Exact mass from MS data | Mascot results | Exact mass calculated from Mascot data | Retention time | Well numbers | Toxin ID | Coverage % | Protein score | Toxin class | Activity |  |
| --- | --- | --- | --- | --- | --- | --- | --- | --- | --- | --- | --- |
| *Daboia russelli russelli* | 1115.5759 | - | - |  |  |  | - |  | - | Anticoagulant |  |
|  |  |  |  |  |  |  |  |  |  |  |  |
|  | **13587,2248** | **PA2B8_DABRR** | **13587,20270** | 13.95  17.04  18.36 | B8  F10  G11 | 1 | 19  82  62 | 74  1248  887 | PLA2 | Neurotoxic/Anticoagulant |  |
|  | 13649,2513 | - | - |  |  |  | - |  |  | Anticoagulant |  |
|  | - | PA2BS_DABSI (*Daboia siamensis*) | - | 15.05 | I9 | 2 | 78.51 | 882 | PLA2 | Neurotoxic |  |
|  | - | **PA2B5_DABRR** | - | 15.05  17.04  18.36 | I9  F10  G11 | 3 | 41  61  62 | 445  2747  3000 | PLA2 | catalyzes the calcium-dependent hydrolysis of the 2-acyl groups in 3-sn-phosphoglycerides |  |
|  | - | PA2A7_DABSI (*Daboia siamensis*) | - | 17.04 | F10 | 4 | 56.55 | 690 | PLA2 | targets the presynaptic sites of the neuromuscular junction |  |
|  | - | **PA2B3_DABRR** | 13663,179 | 13.62  18.80  19.13  19.35 | E8  K11  N11  P11 | 5 | 49  82  76  47 | 688  1856  1414  335 | PLA2 | catalyzes the calcium-dependent hydrolysis of the 2-acyl groups in 3-sn-phosphoglycerides |  |
|  |  | PA2B_VIPRE (*Vipera renardi*) | - | 17.04  18.36 | F10  G11 | 6 | 33.6  33.6 | 1801  1901 | PLA2 | Anticoagulant |  |
|  | 22778.9073 | - | - |  |  |  | - |  | - | Procoagulant |  |
|  |  | **TXVE_DABRR** | - | 13.62  13.95 | E8  B8 | 7 | 52.7  27.7 | 231  181 | Snake venom vascular endothelial growth factor | Induces capillary permeability and angiogenesis |  |
|  | - | TXVE2_MACLB (*Macrovipera lebetina*) | - | 13.62  13.95 | E8  B8 | 8 | 14  7 | 50  50 | Snake venom vascular endothelial growth factor | Induces capillary permeability and angiogenesis |  |
|  | - | VM3CX_DABSI (*Daboia siamensis*) | - | 13.62  19.13  19.35  19.69  20.13  20.46 | E8  N11  P11  N12  J12  G12 | 9 | 6.28  7.7  12.8  12.8  12.8  7.4 | 55  98  216  146  176  110 | Coagulation factor X-activating enzyme | Procoagulant |  |
|  | - | VM3V3_AGKPL (*Agkistrodon piscivorus leucostoma*) | - | 13.62 | E8 | 10 | 6.28 | 42 | Zinc metalloproteinase | Procoagulant |  |
|  | - | CRVP_AGKPI (*Agkistrodon piscivorus*) | - | 17.04 | F10 | 11 | 4.5 | 50 | Cysteine-rich venom protein | Weakly blocks contraction of smooth muscle |  |
|  | - | **NGFV_DABRR** | - | 15.05 | I9 | 12 | 47 | 199 | Nerve growth factor | development and maintenance of the sympathetic and sensory nervous systems |  |
|  | - | VSP2_MACLB (*Macrovipera lebetina*) | - | 17.04 | F10 | 13 | 21.19 | 113 | Serine proteinase | Not known |  |
|  | - | PA2BA_PROFL (*Protobothrops flavoviridis*) | - | 17.04 | F10 | 14 | 5 | 41 | PLA2 | Edema-inducing activities |  |
|  | - | VSP1_BITGA (*Bitis gabonica*) | - | 17.04 | F10 | 15 | 10.6 | 89 | Serine proteinase | Procoagulant |  |
|  | - | VSPAF_DABSI (*Daboia siamensis*) | - | 17.04 | F10 | 16 | 16.24 | 33 | Fibrinogenase | Caseinolytic activity |  |
|  | - | VSPB_DABSI (*Daboia siamensis*) | - | 18.36  18.80  19.35 | G11  K11  P11 | 17 | 28.9  12.5  12.5 | 982  344  121 | Serine protease | Procoagulant |  |
|  | - | SLA_DABSI (*Daboia siamensis*) | - | 18.36  18.80 | G11  K11 | 18 | 55.73  29.8 | 508  260 | C-type lectin | Anticoagulant |  |
|  | - | SL7_DABSI (*Daboia siamensis*) | - | 18.36  18.80 | G11  K11 | 19 | 33.9  18.9 | 195  127 | C-type lectin | Not known |  |
|  | - | VSP1_MACLB (*Macrovipera lebetina*) | - | 18.80  19.13 | K11  N11 | 20 | 32.2  14.9 | 414  159 | Serine protease | Not known |  |
|  | - | SLA_DABPA (*Daboia palaestinae*) | - | 18.80 | K11 | 21 | 11 | 22 | C-type Lectin | Not known |  |
|  | - | V5NTD_GLOBR (*Gloydius brevicaudus*) | - | 19.13 | N11 | 22 | 7.8 | 178 | Snake venom 5'-nucleotidase | Hydrolyzes nucleotides into nucleosides |  |
|  | - | VSPY_MACLB (*Macrovipera lebetina*) | - | 19.13 | N11 | 23 | 11.6 | 75 | Serine protease | Procoagulant |  |
|  | - | SLLC1_DABSI (*Daboia siamensis*) | - | 19.35 | P11 | 24 | 13.8 | 69 | C-type lectin | Procoagulant |  |
|  | - | SLLC2_MACLB (*Macrovipera lebetina*) | - | 19.35  20.13  20.46 | P11  J12  G12 | 25 | 5.9  13  8.1 | 57  66  57 | C-type lectin | Procoagulant |  |
|  | - | V5NTD_CROAD (*Crotalus adamanteus*) | - | 19.35 | P11 | 26 | 2.3 | 57 | Snake venom 5'-nucleotidase | Hydrolyzes nucleotides into nucleosides |  |
|  | - | SL4_DABSI (*Daboia siamensis*) | - | 20.13 | J12 | 27 | 8 | 131 | C-type lectin | Not known |  |
|  | - | SLCIB_MACLB (*Macrovipera lebetina*) | - | 20.13 | J12 | 28 | 13 | 43 | C-type lectin | Not known |  |
|  | - | VSPBF_MACLB  (*Macrovipera lebetina*) | - | 20.13  20.46  21.23  22.22 | J12  G12  A13  J13 | 29 | 5.6  5.6  5.6  5.6 | 32  37  17  17 | Serine protease | Procoagulant |  |
|  | - | SL3_DABSI (*Daboia siamensis*) | - | 20.46 | G12 | 30 | 33.6 | 543 | C-type lectin | Not known |  |
|  | - | SLAE_MACLB (*Macrovipera lebetina*) | - | 20.46 | G12 | 31 | 29.5 | 246 | C-type lectin | Not known |  |
|  | - | **OXLA_DABRR** | - | 19.35  19.69 | P11  N12 | 32 | 52.9  29.4 | 2484  500 |  | Endema, myofibrosis, and inflammatory cell infiltration |  |
|  |  |  |  |  |  |  |  |  |  |  |  |
| *Bothrops asper* | 13765.5812 | **PA2H3_BOTAS** | 13765.58896 | 13.95  14.17  14.39  14.94  15.27  15.61  16.16 | B8  A9  C9  H9  K9  N9  N10 | 1 | 79.5  79.5  78.7  62.3  53.7  39.6  42.6 | 2558  5456  6176  440  163  111  177 | PLA2 | Myotoxic/Anticoagulant |  |
|  | 13714.5646 | **PA2H2_BOTAS** | 13714.56817 | 13.95  14.17  14.39  14.94  15.27  15.61  17.59 | B8  A9  C9  H9  K9  N9  A10 | 2 | 81.1  81.1  81.1  50.4  42.9  28.1  13.2 | 758  3378  3429  305  95  47  57 | PLA2 | Myotoxic/Anticoagulant |  |
|  | 13957.5333 | **PA2B3_BOTAS** | 13957.48720 | 16.16  16.38  16.93 | N10  L10  G10 | 3 | 53.3  74.8  83.7 | 185  648  2597 | PLA2 | Myotoxic/Anticoagulant |  |
|  | 13925.5802 | - | - |  |  |  |  |  | - | Anticoagulant |  |
|  | 799.4155 | - | - |  |  |  |  |  | - | Anticoagulant |  |
|  | 13912.4649 | - | - |  |  |  |  |  |  | Anticoagulant |  |
|  | 23814.0019 | - | - |  |  |  |  |  | - | Procoagulant |  |
|  | - | **PA2HA_BOTAS** | 13896.51308 | 14.94  15.27  15.61  16.38 | H9  K9  N9  L10 | 4 | 63.6  54.5  35.5  16.5 | 1041  196  134  39 | PLA2 | Myotoxic/Anticoagulant |  |
|  | - | **VM1B1_BOTAS** | 24288.0468 | 17.59  17.70  17.92  18.47  18.69  19.24 | A10  A11  C11  H11  J11  O11 | 5 | 27.1  30  53  24  27  12 | 331  594  1424  684  276  86 | Zinc metalloproteinase | Procoagulant |  |
|  | - | PA2H2_CERGO  (*Cerrophidion godmani*) | - | 14.94 | H9 | 6 | 34 | 131 | PLA2 | Myotoxic |  |
|  | - | NGFV_BOTJR  (*Bothrops jararacussu*) | - | 14.94 | H9 | 7 | 25 | 80 | Venom nerve growth factor | development and maintenance of the sympathetic and sensory nervous systems |  |
|  | - | SLA1_DEIAC (*Deinagkistrodon acutus*) | - | 14.94 | H9 | 8 | 14.5 | 32 | C-type lectin | Procoagulant |  |
|  | - | NGFV_AGKCO (*Agkistrodon contortrix contortrix*) | - | 15.27  15.61  16.16 | K9  N9  N10 | 9 | 8  5  5 | 43  47  36 | Venom nerve growth factor | development and maintenance of the sympathetic and sensory nervous systems |  |
|  | - | PA2B2_BOTJR (*Bothrops jararacussu*) | - | 16.16  16.38 | N10  L10 | 10 | 47.5  68.9 | 154  369 | PLA2 | Hemolytic, anticoagulant, and cytotoxic activities |  |
|  | - | PA2H2_BOTMO (*Bothrops moojeni*) | - | 16.16 | N10 | 11 | 37.7 | 133 | PLA2 | Myotoxin and edema-inducing activities |  |
|  | - | PA2B_BITCA (*Bitis caudalis*) | - | 16.16 | N10 | 12 | 19.8 | 51 | PLA2 | Anticoagulant activity and presynaptic neurotoxicity |  |
|  | - | OXLA_CERCE (*Cerastes cerastes*) | - | 16.16  19.24  19.47  19.58 | N10  O11  P12  O12 | 13 | 1.4  18  15  15 | 50  1048  217  212 | L-amino acid oxidase | Not known |  |
|  | - | PA2BN_CROVV (*Crotalus viridis viridis*) | - | 16.16  16.38  16.93 | N10  L10  G10 | 14 | 11.5  11.5  11.5 | 48  50  47 | PLA2 | Myonecrosis and edema-inducing activity |  |
|  | - | **VSPL_BOTAS** | - | 16.38  16.93  17.59 | L10  G10  A10 | 15 | 9.4  3.8  15.3 | 162  49  191 | Thrombin-like enzyme | Procoagulant |  |
|  | - | VSP17_CRODU (*Crotalus durissus terrificus*) | - | 16.38 | L10 | 16 | 12 | 135 | Thrombin-like enzyme | Procoagulant |  |
|  | - | VSP1_BUNMU (*Bungarus multicinctus*) | - | 16.38 | L10 | 17 | 9.2 | 134 | Serine protease | Not known |  |
|  | - | PA2HB_BOTAL (*Bothrops alternatus*) | - | 16.38 | L10 | 18 | 27.2 | 79 | PLA2 | Myotoxic |  |
|  | - | PA2B3_DABRR (*Daboia russelii*) | - | 16.38 | L10 | 19 | 7.4 | 40 | PLA2 | Not known |  |
|  | - | PA2BD_BOTLC (*Bothrops leucurus*) | - | 16.93 | G10 | 20 | 54 | 1190 | PLA2 | Cytotoxic and anticoagulant |  |
|  | - | CRVP_AGKPI (*Agkistrodon piscivorus piscivorus*) | - | 16.93 | G10 | 21 | 15.4 | 227 | Cysteine-rich venom protein | Blocks contraction of smooth muscle |  |
|  | - | VSP1_BOTJA (*Bothrops jararaca*) | - | 17.59  17.70  17.92  18.47  18.69 | A10  A11  C11  H11  J11 | 22 | 42.7  40  25  25  12 | 1079  2250  1252  355  96 | Platelet-aggregating proteinase | Procoagulant |  |
|  | - | **PA2A2_BOTAS** | - | 17.59  17.70  17.92 | A10  A11  C11 | 23 | 77.4  80  28 | 1008  976  60 | PLA2 | No activity |  |
|  | - | VM1B2_BOTJR (*Bothrops jararacussu*) | - | 17.59  17.70  18.47  18.69 | A10  A11  H11  J11 | 24 | 16.5  16  13  18 | 319  830  661  264 | Metalloproteinase | Procoagulant |  |
|  | - | **VM2_BOTAS** | - | 17.59  17.70  18.47  18.69  19.24  19.58  20.68 | A10  A11  H11  J11  O11  O12  E12 | 25 | 15  20  12  17  7  12  9 | 128  174  161  105  85  219  96 | Zinc metalloproteinase | Anticoagulant |  |
|  | - | PA2A_BOTJR (*Bothrops jararacussu*) | - | 17.59 | A10 | 26 | 32 | 63 | PLA2 | Edema and anticoagulant |  |
|  | - | PA2B1_LACMU (*Lachesis muta muta*) | - | 17.59 | A10 | 27 | 15 | 58 | PLA2 | Neurotoxic and myotoxic |  |
|  | - | VSPPA_GLOBR (*Gloydius brevicaudus*) | - | 17.59 | A10 | 28 | 8.1 | 26 | Venom plasminogen activator | Anticoagulant |  |
|  | - | VSP14_BOTJA (*Bothrops jararaca*) | - | 17.70  17.92  18.47  18.69  19.24 | A11  C11  H11  J11  O11 | 29 | 33  39  11  12  14 | 536  1490  151  57  129 | Snake venom serine protease | Not known |  |
|  | - | VSPA_BOTJA (*Bothrops jararaca*) | - | 17.70  17.92  18.47  18.69 | A11  C11  H11  J11 | 30 | 22  40  32  25 | 182  367  384  137 | Thrombin-like enzyme | Procoagulant |  |
|  | - | CRVP_PROMU (*Protobothrops mucrosquamatus*) | - | 16.93 | G10 | 31 | 16.3 | 183 | Cysteine-rich venom protein | Not known |  |
|  | - | VM1B1_BOTPA (*Bothrops pauloensis*) | - | 17.70  17.92  19.47 | A11  C11  P12 | 32 | 33  40  10 | 115  122  51 | Metalloproteinase | Myotoxic and procoagulant |  |
|  | - | VSPD_GLOUS (*Gloydius ussuriensis*) | - | 17.70 | A11 | 33 | 10 | 61 | Bradykinin-releasing enzyme | Anticoagulant |  |
|  | - | VM1BI_BOTMO (*Bothrops moojeni*) | - | 17.92 | C11 | 34 | 24 | 2861 | Metalloproteinase | Anticoagulant |  |
|  | - | VSPL_BOTLC (*Bothrops leucurus*) | - | 17.92  18.47 | C11  H11 | 35 | 48  34 | 401  456 | Thrombin-like enzyme | Procoaglant |  |
|  | - | VSPPA_AGKPL (*Agkistrodon piscivorus leucostoma*) | - | 17.92 | C11 | 36 | 18 | 369 | Venom plasminogen activator | Anticoagulant |  |
|  | - | VSP_LACST (*Lachesis stenophrys*) | - | 18.47 | H11 | 37 | 25 | 242 | Serine protease | Not known |  |
|  | - | VSPB_DABSI (*Daboia siamensis*) | - | 18.47 | H11 | 38 | 16 | 212 | Beta-fibrinogenase-like | Fibrinogenolytic |  |
|  | - | SLEB_BOTJA (*Bothrops jararaca*) | - | 18.47 | H11 | 39 | 12 | 90 | C-type lectin | Procoagulant |  |
|  | - | **SLA_BOTAS** | - | 18.47 | H11 | 40 | 17 | 47 | C-type lectin | Procoagulant |  |
|  | - | V5NTD_GLOBR (*Gloydius brevicaudus*) | - | 18.69 | J11 | 41 | 9 | 169 | Snake venom 5'-nucleotidase | Not known |  |
|  | - | VSPF_BOTAT (*Bothrops atrox*) | - | 18.69 | J11 | 42 | 23 | 99 | Thrombin-like enzyme | Procoagulant |  |
|  | - | OXLA_BOTMO (*Bothrops moojeni*) | - | 19.24  19.47  19.58 | O11  P12  O12 | 43 | 62  46  44 | 2305  678  922 | L-amino-acid oxidase | Not known |  |
|  | - | OXLA_BOTPA (*Bothrops pauloensis*) | - | 19.24  19.47  19.58 | O11  P12  O12 | 44 | 45  36  36 | 1648  417  683 | L-amino-acid oxidase | Not known |  |
|  | - | OXLA2_CROAD (*Crotalus adamanteus*) | - | 19.24  19.47  19.58 | O11  P12  O12 | 45 | 27  22  23 | 891  293  417 | L-amino-acid oxidase | Not known |  |
|  | - | OXLA_SISCA (*Sistrurus catenatus edwardsii*) | - | 19.24  19.47  19.58 | O11  P12  O12 | 46 | 23  17  19 | 613  157  254 | L-amino-acid oxidase | Not known |  |
|  | - | VM31_BOTAT (*Bothrops atrox*) | - | 19.24  19.47  19.58 | O11  P12  O12 | 47 | 7  12  17 | 155  504  576 | Zinc metalloproteinase-disintegrin | Procoagulant |  |
|  | - | OXLA_CRODM (*Crotalus durissus cumanensis*) | - | 19.47  19.58 | P12  O12 | 48 | 17  18 | 198  262 | L-amino acid oxidase | Not known |  |
|  | - | PDE2_CROAD (*Crotalus adamanteus*) | - | 19.47  19.58 | P12  O12 | 49 | 8  9 | 173  298 | Venom phosphodiesterase | Anticoagulant |  |
|  | - | VM3V1_CROAT (*Crotalus atrox*) | - | 19.24 | O11 | 50 | 6 | 122 | Zinc metalloproteinase-disintegrin | Procoagulant |  |
|  | - | SLCB_DEIAC (*Deinagkistrodon acutus*) | - | 20.68  21.12  22.33 | E12  A12  K13 | 51 | 6  6  6 | 43  51  41 | C-type lectin | No activity |  |
|  | - | VM33_BOTAT (*Bothrops atrox*) | - | 22.00 | H13 | 52 | 5 | 33 | Zinc metalloproteinase-disintegrin | Procoagulant |  |
|  | - | VSPBF_MACLB (*Macrovipera lebetina*) | - | 22.00  22.33 | H13  K13 | 53 | 6  6 | 24  32 | Beta-fibrinogenase | Fibrinogenolytic |  |
|  | - | VM3AA_CROAT (*Crotalus atrox*) | - | 22.33 | K13 | 54 | 4 | 88 | Zinc metalloproteinase-disintegrin | Procoagulant |  |
|  |  | VM3JA_BOTJA |  | 19.24 | O11 | 55 | 5 | 61 |  |  |  |
|  |  | VM33_CROAD |  | 19.24 | O11 | 56 | 4 | 60 |  |  |  |
|  |  | VM3_BUNFA |  | 19.47  19.58 | P12  O12 | 57 | 3  3 | 78  82 |  |  |  |
|  |  | VM2TA_TRIGA |  | 19.47  19.58 | P12  O12 | 58 | 7  7 | 36  42 |  |  |  |
|  |  |  |  |  |  |  |  |  |  |  |  |
| *Calloselasma rhodostoma* | 13665,0848 | **PA2BD_CALRH** | 13665,0237 | 15.72  15.94  16.60  17.04 | O9  P10  J10  F10 | 1 | 87  87  50  33 | 1107  1297  211  107 | Inactive basic phospholipase A2 | Anticoagulant |  |
|  | 27872.3544 | - | - |  |  |  |  |  | - | Procoagulant |  |
|  | 21881.1683 | - | - |  |  |  |  |  | - | Procoagulant |  |
|  | 22693.1304 | - | - |  |  |  |  |  | - | Procoagulant |  |
|  | - | VM2B1_AGKBI (*Agkistrodon bilineatus*) | - | 15.72  16.60 | O9  J10 | 2 | 3  3 | 79  100 | Zinc metalloproteinase-disintegrin | Procoagulant |  |
|  | - | **VSPF1_CALRH** | - | 16.60  17.04  17.37  18.25 | J10  F10  C10  F11 | 3 | 58  44  25  31 | 3658  1520  87  229 | Thrombin-like enzyme | Anticoagulant |  |
|  | - | **SLEA_CALRH** | - | 16.60  17.04  17.37 | J10  F10  C10 | 4 | 33  68  38 | 798  3094  213 | C-type lectin | Anticoagulant |  |
|  | - | **SLEB_CALRH** | - | 16.60  17.04  20.57 | J10  F10  F12 | 5 | 43  7  6 | 201  57  47 | C-type lectin | Anticoagulant |  |
|  | - | **VSPF2_CALRH** | - | 17.04  17.37  18.03  18.25  18.58 | F10  C10  D11  F11  I11 | 6 | 20  14  36  53  22 | 247  737  409  952  97 | Thrombin-like enzyme | Procoagulant |  |
|  | - | PA2AD_TRIST (*Trimeresurus stejnegeri*) | - | 17.04  17.37  18.03 | F10  C10  D11 | 7 | 19  73  22 | 59  352  50 | PLA2 | Not known |  |
|  | - | PA2AA_CROVV (*Crotalus viridis viridis*) | - | 17.37 | C10 | 8 | 17 | 61 | PLA2 | Anticoagulant |  |
|  | - | **PA2AB_CALRH** | - | 18.03 | D11 | 9 | 83 | 1315 | PLA2 | Anticoagulant |  |
|  | - | **SLYA_CALRH** | - | 18.03  18.25 | D11  F11 | 10 | 15  53 | 153  317 | C-type lectin | Procoagulant |  |
|  | - | **SLYB_CALRH** | - | 18.25 | F11 | 11 | 15 | 39 | C-type lectin | Procoagulant |  |
|  | - | VSP14_BOTJA (*Bothrops jararaca*) | - | 18.25 | F11 | 12 | 14 | 289 | Serine protease | Not known |  |
|  | - | VM1B2_BOTJR (*Bothrops jararacussu*) | - | 18.25 | F11 | 13 | 8.5 | 111 | Metalloproteinase | Anticoagulant |  |
|  | - | **VM1K_CALRH** | - | 18.25  20.02  20.57  20.90  21.34 | F11  K12  F12  C12  B13 | 14 | 12  33  33  17  27 | 41  607  252  51  64 | Metalloproteinase kistomin | Anticoagulant |  |
|  | - | VM1B1_BOTPA (*Bothrops pauloensis*) | - | 18.25 | F11 | 15 | 10 | 39 | Metalloproteinase | Cell detachment and a apoptosis (anoikis) in endothelial cells |  |
|  | - | VSPBF_MACLB (*Macrovipera lebetina*) | - | 18.58 | I11 | 16 | 6 | 38 | Beta-fibrinogenase | Anticoagulant |  |
|  | - | **OXLA_CALRH** | - | 19.02  20.02  20.57  20.90  21.34  21.67  21.89  22.11  22.88 | M11  K12  F12  C12  B13  E13  G13  I13  P13 | 17 | 3  18  18  13  13  2  3  7  6 | 104  402  314  125  197  39  44  52  48 | L-amino-acid oxidase | Not known |  |
|  | - | **VM2RH_CALRH** | - | 20.02  20.57  20.90  21.34 | K12  F12  C12  B13 | 18 | 27  20  5  4 | 344  153  73  106 | Zinc metalloproteinase/disintegrin | Anticoagulant |  |
|  | - | VM2_BOTAS (*Bothrops asper*) | - | 20.57 | F12 | 19 | 9 | 66 | Zinc metalloproteinase/disintegrin | Anticoagulant |  |
|  |  | VM1B1_BOTAS |  | 18.25 | F11 | 20 | 6 | 102 |  |  |  |
|  |  |  |  |  |  |  |  |  |  |  |  |
| *Oxyuranus scutelatus* | 6993.2858 | **VKT_OXYSC** | 6993.2944 | 11.08  12.30  12.74  13.07  13.51 | E7  P7  M8  J8  F8 | 1 | 30  87  84  57  24 | 45  1250  684  99  60 | Kunitz-type serine protease inhibitor taicotoxin | Anticoagulant |  |
|  | 6536.8065 | **VKT3_OXYSC** | 6536.8236 | 14.17 | A9 | 2 | 91 | 452 | Kunitz-type serine protease inhibitor scutellin | Anticoagulant |  |
|  | 5737.5820 | - | - |  |  |  |  |  | - | Anticoagulant |  |
|  | 7090.3244 | - | - |  |  |  |  |  | - | Anticoagulant |  |
|  | 6993.2944 | - | - |  |  |  |  |  | - | Anticoagulant |  |
|  | 13151.3900 | - | - |  |  |  |  |  | - | Anticoagulant |  |
|  | 13600.7370 | - | - |  |  |  |  |  | - | Anticoagulant |  |
|  | 14052.0601 | - | - |  |  |  |  |  | - | Anticoagulant |  |
|  | 13222.6761 | - | - |  |  |  |  |  | - | Anticoagulant |  |
|  | 6536.8236 | - | - |  |  |  |  |  | - | Anticoagulant |  |
|  | 21023.533 | - | - |  |  |  |  |  | - | Procoagulant |  |
|  | 22695.5412 | - | - |  |  |  |  |  | - | Procoagulant |  |
|  | - | 3L2O2_OXYMI (*Oxyuranus microlepidotus*) | - | 11.08  12.30 | E7  P7 | 3 | 71  71 | 216  295 | Long neurotoxin 3FTx-Oxy2 | Neurotoxic |  |
|  | - | 3L21_OXYMI (*Oxyuranus microlepidotus*) | - | 11.08  12.30  13.51 | E7  P7  F8 | 4 | 53  65  10 | 210  274  41 | Long neurotoxin | Neurotoxic |  |
|  | - | **3S11_OXYSC** | - | 11.08 | E7 | 5 | 21 | 199 | Short neurotoxin | Neurotoxic |  |
|  | - | 3L21_BUNCA (*Bungarus candidus*) | - | 11.08 | E7 | 6 | 14 | 67 | Alpha-elapitoxin-Bc2a | Neurotoxic |  |
|  | - | PA2A1_OPHHA | - | 12.30  12.74  13.07  13.51  14.17 | P7  M8  J8  F8  A9 | 7 | 9  9  9  9  9 | 73  160  126  139  108 | Acidic phospholipase A2 | Cardiotoxicity, myotoxicity, antiplatelet activity, and edema-inducing activity |  |
|  | - | **PA2TA_OXYSC** | - | 12.74  13.51  13.84  14.17  17.15 | M8  F8  C8  A9  E10 | 8 | 18  56  56  47  21 | 44  427  543  237  98 | Basic phospholipase A2 taipoxin | Neurotoxic |  |
|  | - | **PA2TB_OXYSC** | - | 13.07  14.72  15.05  15.27  16.27 | J8  F9  I9  K9  M10 | 9 | 87  74  87  87  34 | 1116  434  955  900  94 | Neutral phospholipase A2 homolog taipoxin | Neurotoxic |  |
|  | - | PA2PA_OXYMI (*Oxyuranus microlepidotus*) | - | 13.51  13.84  14.17 | F8  C8  A9 | 10 | 28  28  21 | 191  261  149 | Basic phospholipase A2 | Neurotoxic |  |
|  | - | **PA21_OXYSC** | - | 14.72  15.05  15.27  15.83  16.27  16.60  16.93  17.15 | F9  I9  K9  P9  M10  J10  G10  E10 | 11 | 76  74  78  86  78  74  58  27 | 1828  1053  3405  9792  3367  861  306  54 | Phospholipase A2 | Triggers cell migration, antimalarial activity |  |
|  | - | **PA2TC_OXYSC** | - | 14.72  15.05  15.27  16.27 | F9  I9  K9  M10 | 12 | 64  65  65  19 | 1107  2827  386  76 | Neutral phospholipase A2 | Neurotoxic |  |
|  | - | PA2PB_OXYMI (*Oxyuranus microlepidotus*) | - | 14.72  15.05 | F9  I9 | 13 | 36  68 | 278  832 | Neutral phospholipase A2 | Neurotoxic |  |
|  | - | **NGFV_OXYSC** | - | 14.72  15.05  15.27  16.93 | F9  I9  K9  G10 | 14 | 39  56  39  12 | 267  280  220  51 | Venom nerve growth factor | Development and maintenance of the sympathetic and sensory nervous systems. Metalloproteinase inhibitor. |  |
|  | - | VNPA_OXYMI (*Oxyuranus microlepidotus*) | - | 14.72  15.05  15.27  16.27 | F9  I9  K9  M10 | 15 | 65  60  65  68 | 152  88  122  45 | Natriuretic peptide TNP-a | Exhibits vasoactivenand hypotensive activity |  |
|  | - | PA2N3_BUNFA (*Bungarus fasciatus*) | - | 14.72 | F9 | 16 | 16 | 71 | Neutral phospholipase A2 | Not known |  |
|  | - | PA2CC_OXYSA (*Oxyuranus scutellatus canni*) | - | 15.05 | I9 | 17 | 87 | 2294 | Neutral phospholipase A2 homolog cannitoxin | Neurotoxic |  |
|  | - | PA2HB_MICTN (*Micrurus tener tener*) | - | 15.27 | K9 | 18 | 8 | 46 | Basic phospholipase A2 | Neurotoxic |  |
|  | - | **3SX3_OXYSC** | - | 15.27  16.93 | K9  G10 | 19 | 17  17 | 35  33 | Short neurotoxin | Neurotoxic |  |
|  | - | VNPA_PSEAU (*Pseudechis australis*) | - | 15.83  16.27  16.60  16.93  17.15 | P9  M10  J10  G10  E10 | 20 | 60  60  90  90  27 | 77  87  162  306  92 | Natriuretic peptide PaNP-a | Hypotensive and vasodepressor |  |
|  | - | 3SX6_OXYMI (*Oxyuranus microlepidotus*) | - | 16.60  16.93 | J10  G10 | 21 | 32  31 | 221  47 | Toxin 3FTx-Oxy6 | Neurotoxic |  |
|  | - | VSPF1_CALRH (*Calloselasma rhodostoma*) | - | 16.93  17.15 | G10  E10 | 22 | 44  20 | 617  85 | Thrombin-like enzyme ancrod | Anticoagulant |  |
|  | - | CYT_OXYMI (*Oxyuranus microlepidotus*) | - | 16.93 | G10 | 23 | 7 | 45 | Cystatin | Not known |  |
|  | - | SLEA_CALRH (*Calloselasma rhodostoma*) | - | 17.15 | E10 | 24 | 42 | 95 | C-type lectin | Anticoagulant |  |
|  | - | OXLA_CALRH (*CalloselaCR1sma rhodostoma*) | - | 19.35  20.24 | P11  I12 | 25 | 16  16 | 208  208 | L-amino-acid oxidase | Not known |  |
|  |  |  |  |  |  |  |  |  |  |  |  |
| *Echis ocellatus* | 13815.3523 | **PA2HS_ECHOC** | 13815,2798 | 13.95 | B8 | 1 | 81 | 1454 | PLA2 | Anticoagulant |  |
|  | 13856.1382 | **PA2A5_ECHOC** | 13856,0665 | 15.49  16.16 | M9  N10 | 2 | 63  57 | 6801  448 | Acidic phospholipase A2 | Not known |  |
|  | 14030.1579 | - | - |  |  |  |  |  | - | Anticoagulant |  |
|  | 52015.873 | - | - |  |  |  |  |  | - | Procoagulant |  |
|  | - | **OXLA_ECHOC** | - | 13.95  19.47 | B8  P12 | 3 | 2  7 | 136  57 | L-amino-acid oxidase | Not known |  |
|  | - | VSP1_MACLB (*Macrovipera lebetina*) | - | 19.47  19.80 | P12  M12 | 4 | 10  10 | 64  193 | Serine protease | Not known |  |
|  | - | VM1F_MACLB (*Macrovipera lebetina*) | - | 19.47 | P12 | 5 | 4 | 39 | metalloproteinase fibrolase | Anticoagulant |  |
|  | - | **VM3E2_ECHOC** | - | 13.95  19.80  20.13 | B8  M12  J12 | 6 | 5  48  45 | 55  1683  1248 | Zinc metalloproteinase-disintegrin | Procoagulant |  |
|  | - | VM3BE_BOTER (*Bothrops erythromelas*) | - | 19.80  20.13 | M12  J12 | 7 | 3  3 | 398  274 | Zinc metalloproteinase-disintegrin | Procoagulant |  |
|  | - | **SL1_ECHOC** | - | 19.80  20.13 | M12  J12 | 8 | 53  37 | 275  191 | C-type lectin | Not known |  |
|  | - | VM2AL_TRIAB (*Trimeresurus albolabris*) | - | 19.80 | M12 | 9 | 7 | 190 | Zinc metalloproteinase homolog-disintegrin | Not known |  |
|  | - | **SL124_ECHOC** | - | 19.80  20.13  20.57 | M12  J12  F12 | 10 | 31  21  20 | 70  72  37 | C-type lectin | Not known |  |
|  | - | SL2_BITGA (*Bitis gabonica*) | - | 19.80  20.13 | M12  J12 | 11 | 12  20 | 45  69 | C-type lectin | Not known |  |
|  | - | VSPGL_GLOSH (*Gloydius shedaoensis*) | - | 19.80 | M12 | 12 | 6 | 37 | Thrombin-like enzyme | Procoagulant, amidolytic |  |
|  | - | VM25A_BITAR (*Bitis arietans*) | - | 20.13  20.57 | J12  F12 | 13 | 37  21 | 918  494 | Zinc metalloproteinase-disintegrin | Procoagulant |  |
|  | - | **VM3E6_ECHOC** | - | 20.13  20.57 | J12  F12 | 14 | 23  11 | 126  31 | Zinc metalloproteinase-disintegrin | Procoagulant |  |
|  | - | SLAE_MACLB (*Macrovipera lebetina*) | - | 20.13 | J12 | 15 | 27 | 89 | C-type lectin | Not known |  |
|  | - | VM3AA_CROAT (*Crotalus atrox*) | - | 20.13 | J12 | 16 | 3 | 52 | Zinc metalloproteinase-disintegrin | Procoagulant |  |
|  | - | VSP1_BUNMU (*Bungarus multicinctus*) | - | 20.13 | J12 | 17 | 6 | 36 | Serine protease | Not known |  |
|  | - | VM3CX_MACLB (*Macrovipera lebetina*) | - | 20.57 | F12 | 18 | 3 | 37 | Coagulation factor X-activating enzyme | Procoagulant |  |
|  | - | VM3LC_MACLN *(Macrovipera lebetina transmediterranea*) | - | 21.12 | A12 | 19 | 10 | 141 | Disintegrin-like leberagin-C | Anticoagulant |  |
|  | - | VM3HB_PROFL *(Protobothrops flavoviridis*) | - | 21.12 | A12 | 20 | 3 | 52 | Zinc metalloproteinase-disintegrin | Procoagulant |  |
|  | - | VM34_DRYCN (*Drysdalia coronoides*) | - | 21.12 | A12 | 21 | 3 | 33 | Zinc metalloproteinase-disintegrin | Not known |  |
|  |  |  |  |  |  |  |  |  |  |  |  |
| *Bothrops jararaca* | 872.3494 | - | - |  |  |  |  |  | - | Anticoagulant |  |
|  | 1369.6846 | - | - |  |  |  |  |  | - | Anticoagulant |  |
|  | 412.1175 | - | - |  |  |  |  |  | - | Anticoagulant |  |
|  | 733.4422 | - | - |  |  |  |  |  | - | Anticoagulant |  |
|  | 24665.602 | - | - |  |  |  |  |  | - | Procoagulant |  |
|  | 26531.502 | - | - |  |  |  |  |  | - | Procoagulant |  |
|  | 23013.547 | - | - |  |  |  |  |  | - | Procoagulant |  |
|  | 79714.443 | - | - |  |  |  |  |  | - | Procoagulant |  |
|  | - | **VM3JA_BOTJA** | - | 13.95  21.89  22.22  23.99 | B8  G13  J13  G14 | 1 | 18  23  46  11 | 518  290  3285  1480 | Zinc metalloproteinase-disintegrin | Procoagulant |  |
|  | - | PA2B1_BOTJR (*Bothrops jararacussu*) | - | 13.95  14.94 | B8  H9 | 2 | 11  18 | 63  48 | Basic phospholipase A2 | Muscle necrosis, edema |  |
|  | - | **TXVE_BOTJA** | - | 13.95  14.94  15.61  16.49  17.04  17.48 | B8  H9  N9  K10  F10  B10 | 3 | 16  39  45  19  16  16 | 43  376  878  47  108  74 | Snake venom vascular endothelial growth factor | Capillary permeability and angiogenesis |  |
|  | - | TXVE_BOTIN (*Bothrops insularis*) | - | 14.94  15.61 | H9  N9 | 4 | 19  19 | 163  138 | Snake venom vascular endothelial growth factor | Capillary permeability and angiogenesis |  |
|  | - | PA2H2_BOTMO (*Bothrops moojeni*) | - | 14.94  15.61 | H9  N9 | 5 | 24  49 | 54  240 | Basic phospholipase A2 | Myotoxin and edema-inducing |  |
|  | - | VM1AH_AGKCL (*Agkistrodon contortrix laticinctus*) | - | 14.94 | H9 | 6 | 3 | 50 | metalloproteinase | Procoagulant |  |
|  | - | **BPP11_BOTJA** | - | 14.94 | H9 | 7 | 54 | 33 | Bradykininpotentiating peptide | hypotensive agent |  |
|  | - | NGFV_BOTJR (*Bothrops jararacussu*) | - | 15.61 | N9 | 8 | 28 | 83 | Venom nerve growth factor | Development and maintenance of the sympathetic and sensory nervous systems |  |
|  | - | CRVP_CROAD (*Crotalus adamanteus*) | - | 16.49 | K10 | 9 | 25 | 523 | Cysteine-rich secretory protein | Blocks contraction of smooth muscle |  |
|  | - | CRVP_PROMU (*Protobothrops mucrosquamatus*) | - | 16.49 | K10 | 10 | 17 | 269 | Cysteine-rich venom protein | Not known |  |
|  | - | VSPB_TRIGA (*Trimeresurus gramineus*) | - | 16.49 | K10 | 11 | 4 | 77 | Serine protease | Not known |  |
|  | - | VSPPA_TRIAB (*Trimeresurus albolabris*) | - | 16.49 | K10 | 12 | 8 | 64 | Plasminogen activator | Anticoagulant |  |
|  | - | VSP1_DEIAC (*Deinagkistrodon acutus*) | - | 16.49 | K10 | 13 | 6 | 37 | Thrombin-like enzyme acutobin | Procoagulant |  |
|  | - | CRIS_PROFL (*Protobothrops flavoviridis*) | - | 16.49 | K10 | 14 | 10 | 20 | Serotriflin | Not known |  |
|  | - | LECG_BOTIN (*Bothrops insularis*) | - | 17.04  17.37  17.48  17.59  17.81  18.03 | F10  C10  B10  A10  B11  D11 | 15 | 74  56  56  26  9  15 | 702  434  291  136  62  74 | C-type lectin | Hemagglutinating |  |
|  | - | VSPH_BOTJR (*Bothrops jararacussu*) | - | 17.04  17.37  17.48  17.59  17.81 | F10  C10  B10  A10  B11 | 16 | 47  23  24  17  15 | 549  151  102  76  102 | Serine protease | Not known |  |
|  | - | CRVP_AGKPI (*Agkistrodon piscivorus piscivorus*) | - | 17.04 | F10 | 17 | 4 | 49 | Cysteine-rich venom protein | Blocks contraction of smooth muscle |  |
|  | - | PA2A_BOTIN (*Bothrops insularis*) | - | 17.37  17.48  17.59  18.03 | C10  B10  A10  D11 | 18 | 83  88  52  24 | 381  822  227  47 | PLA2 | Edema, neuromuscular blockade, myonecrosis |  |
|  | - | **PA2A_BOTJA** | - | 18.58 | I11 | 19 | 24 | 22 | PLA2 | Anticoagulant |  |
|  | - | VSP10_TRIST (*Trimeresurus stejnegeri*) | - | 17.37 | C10 | 20 | 14 | 200 | Serine protease | Not known |  |
|  | - | VSPL_BOTAS (*Bothrops asper*) | - | 17.37  17.59  17.81  18.03 | C10  A10  B11  D11 | 21 | 9  26  28  28 | 92  166  111  107 | Thrombin-like enzyme | Procoagulant |  |
|  | - | **VSPA_BOTJA** | - | 17.37  17.48  17.81 | C10  B10  B11 | 22 | 6  12  22 | 71  130  79 | Thrombin-like enzyme | Procoagulant |  |
|  | - | **VSP1_BOTJA** | - | 17.48  17.59  17.81  18.03  18.58 | B10  A10  B11  D11  I11 | 23 | 35  49  49  49  11 | 368  1239  2017  1655  98 | Platelet-aggregating proteinase | Procoagulant |  |
|  | - | VSP1_BOTJR (*Bothrops jararacussu*) | - | 17.48 | B10 | 24 | 12 | 134 | Thrombin-like enzyme | Procoagulant |  |
|  | - | VSPE2_GLOUS (*Gloydius ussuriensis*) | - | 17.48 | B10 | 25 | 12 | 111 | Thrombin-like enzyme | Procoagulant |  |
|  | - | VSP2_PROEL (*Protobothrops elegans*) | - | 17.48  17.59  18.03 | B10  A10  D11 | 26 | 9  12  12 | 90  71  86 | Thrombin-like enzyme | Procoagulant |  |
|  | - | VSP1_GLOUS (*Gloydius ussuriensis*) | - | 17.59  17.81  18.03  18.58 | A10  B11  D11  I11 | 27 | 26  24  21  20 | 188  205  113  92 | Thrombin-like enzyme | Procoagulant |  |
|  | - | VSP_BOTIN (*Bothrops insularis*) | - | 17.59 | A10 | 28 | 21 | 65 | serine protease | Not known |  |
|  | - | **VSP20_BOTJA** | - | 17.81  18.03  18.58 | B11  D11  I11 | 29 | 60  71  21 | 689  793  56 | serine proteinase | Not known |  |
|  | - | **VSP14_BOTJA** | - | 17.81  18.03  18.58  19.24  20.02  20.35  20.90  21.89  22.22  23.99  31.82  33.03 | B11  D11  I11  O11  K12  H12  C12  G13  J13  G14  A19  L19 | 30 | 28  49  46  4  28  37  34  17  9  30  34  25 | 181  864  330  39  100  251  107  225  132  248  175  67 | serine protease | Not known |  |
|  | - | VSP1_BUNMU (*Bungarus multicinctus*) | - | 17.81 | B11 | 31 | 11 | 62 | serine protease | Not known |  |
|  | - | VSP6_PROMU (*Protobothrops mucrosquamatus*) | - | 17.81 | B11 | 32 | 12 | 58 | serine protease serpentokallikrein | Not known |  |
|  | - | VSP5_CROAD (*Crotalus adamanteus*) | - | 18.03 | D11 | 33 | 22 | 224 | Serine proteinase | Not known |  |
|  | - | **VSP12_BOTJA** | - | 18.03  18.58 | D11  I11 | 34 | 34  15 | 126  34 | Serine protease | Not known |  |
|  | - | VSPPA_LACMU (Lachesis muta muta) | - | 18.03 | D11 | 35 | 32 | 124 | Plasminogen activator | Anticoagulant |  |
|  | - | **VSP2_BOTJA** | - | 18.58 | I11 | 36 | 40 | 150 | Thrombin-like enzyme | Procoagulant |  |
|  | - | VSPA_DEIAC (*Deinagkistrodon acutus*) | - | 18.58 | I11 | 37 | 11 | 78 | Thrombin-like enzyme | Procoagulant |  |
|  | - | VSP_CRODD (*Crotalus durissus durissus*) | - | 18.58 | I11 | 38 | 19 | 78 | Serine protease | Not known |  |
|  | - | VSPTL_BOTAL (*Bothrops alternatus*) | - | 18.58 | I11 | 39 | 3 | 43 | Serine protease | Not known |  |
|  | - | **SLEA_BOTJA** | - | 19.02  19.24  20.02  20.35  20.90  31.82 | M11  O11  K12  H12  C12  A19 | 40 | 15  15  20  20  15  20 | 217  75  30  73  69  66 | C-type lectin | Procoagulant |  |
|  | - | OXLA_BOTPA (Bothrops pauloensis) | - | 19.02  19.24  20.02 | M11  O11  K12 | 41 | 11  18  7 | 214  254  41 | L-amino-acid oxidase | Not known |  |
|  | - | **SL1A_BOTJA** | - | 19.02  19.24  20.02  20.35  20.90  21.89  22.22  23.99  31.82 | M11  O11  K12  H12  C12  G13  J13  G14  A19 | 42 | 19  20  24  24  19  24  20  24  24 | 207  198  157  206  219  247  64  144  206 | C-type lectin | Receptor blocker for vWF binding |  |
|  | - | **SL1B_BOTJA** | - | 19.02  19.24  20.02  20.35  20.90  21.89  22.22  23.99  31.82 | M11  O11  K12  H12  C12  G13  J13  G14  A19 | 43 | 13  23  23  23  23  31  23  23  23 | 131  141  173  159  155  208  123  152  153 | C-type lectin | Receptor blocker for vWF binding |  |
|  | - | VM2IA_BOTIN (*Bothrops insularis*) | - | 19.02  19.24  20.02  20.35 | M11  O11  K12  H12 | 44 | 9  16  9  12 | 125  366  421  164 | Zinc metalloproteinase/disintegrin | Anticoagulant |  |
|  | - | VM31_LACMR (*Lachesis muta rhombeata*) | - | 19.02 | M11 | 45 | 2 | 38 | Zinc metalloproteinase/disintegrin | Procoagulant |  |
|  | - | **VM2J2_BOTJA** | - | 19.24  19.47  20.02  20.35 | O11  P12  K12  H12 | 46 | 24  10  28  10 | 297  121  220  74 | Zinc metalloproteinase/disintegrin | Hemagglutinating |  |
|  | - | VM11_BOTAT | - | 19.24 | O11 | 47 | 29 | 52 | Metalloproteinase | Procoagulant |  |
|  | - | VM31_BOTAT (*Bothrops atrox*) | - | 19.24  20.02  20.35 | O11  K12  H12 | 48 | 3  3  3 | 36  33  27 | Zinc metalloproteinase-disintegrin | Procoagulant |  |
|  | - | VM1B1_BOTBA (*Bothrops barnetti*) | - | 19.47  20.02  20.35 | P12  K12  H12 | 49 | 12  40  8 | 58  115  24 | Zinc metalloproteinase | Procoagulant |  |
|  | - | **SLAB_BOTJA** | - | 20.02  20.35  20.90 | K12  H12  C12 | 50 | 9  39  43 | 24  40  25 | C-type lectin | Anticoagulant |  |
|  | - | VSPPA_AGKPL (*Agkistrodon piscivorus leucostoma*) | - | 17.48  20.02  20.35  31.82 | B10  K12  H12  A19 | 51 | 24  17  17  17 | 227  80  94  77 | Venom plasminogen activator | Anticoagulant |  |
|  | - | SLA_BOTIN (*Bothrops insularis*) | - | 20.35  21.89 | H12  G13 | 52 | 21  19 | 250  51 | C-type lectin | Anticoagulant |  |
|  | - | OXLA_BOTJR (*Bothrops jararacussu*) | - | 20.35 | H12 | 53 | 3 | 41 | L-amino-acid oxidase | Not known |  |
|  | - | **VM2JR_BOTJA** | - | 20.90 | C12 | 54 | 16 | 184 | Zinc metalloproteinase-disintegrin | Anticoagulant |  |
|  | - | VM36A_BOTIN (*Bothrops insularis*) | - | 21.89  22.22  23.99  31.82  33.03 | G13  J13  G14  A19  L19 | 55 | 22  54  15  12  6 | 151  4570  1482  1249  1023 | Zinc metalloproteinase-disintegrin | Not known |  |
|  | - | **VM3B2_BOTJA** | - | 21.89 | G13 | 56 | 4 | 112 | Zinc metalloproteinase-disintegrin- | Anticoagulant |  |
|  | - | **VM3H3_BOTJA** | - | 21.89 | G13 | 57 | 3 | 102 | Zinc metalloproteinase-disintegrin | Procoagulant |  |
|  | - | VM33_BOTAT (*Bothrops atrox*) | - | 21.89 | G13 | 58 | 3 | 47 | Zinc metalloproteinase-disintegrin | Procoagulant |  |
|  | - | VM3A_BOTAL | - | 32.70 | I19 | 59 | 8 | 934 | Zinc metalloproteinase-disintegrin | Procoagulant |  |
|  |  | **PA2A_BOTJR** |  | 19.02 | M11 | 60 | 20 | 62 |  | Anticoagulant |  |
|  |  | VM1B1_BOTAS |  | 31.82 | A19 | 61 | 3 | 50 |  |  |  |
|  |  | VM1K_CALRH |  | 31.82 | A19 | 62 | 3 | 48 |  |  |  |
|  |  |  |  |  |  |  |  |  |  |  |  |
| *Dispholidus typus* | 13634.0965 | - | - |  |  |  |  |  | - | Anticoagulant |  |
|  | 8432.8015 | - | - |  |  |  |  |  | - | Anticoagulant |  |
|  | 22993,9924 | - | - |  |  |  |  |  | - | Procoagulant |  |
|  | - | **CRVP1_DISTY** | - | 17.92  18.69 | C11  J11 | 1 | 69  8 | 1607  88 | Cysteine-rich venom protein | Blocks contraction of smooth muscle |  |
|  | - | **CRVP3_DISTY** | - | 17.92 | C11 | 2 | 78 | 1291 | Cysteine-rich venom protein | Blocks contraction of smooth muscle |  |
|  | - | CRVP_ECHCO (*Echis coloratus*) | - | 17.92  18.69 | C11  J11 | 3 | 11  4 | 46  62 | Cysteine-rich venom protein | Blocks contraction of smooth muscle |  |
|  |  | VM3A_BOTAL |  | 13.73  14.06  17.26  17.48 | D8  A8  D10  B10 | 4 | 8  8  8  8 | 1120  1137  385  855 |  |  |  |
|  |  | VSP14_BOTJA |  | 17.92  18.69  19.02  20.46 | C11  J11  M11  G12 | 5 | 21  9  9  8 | 157  42  74  32 |  |  |  |
|  |  | VM3BP_BOTJA |  | 18.69  19.02  19.35  22.22  23.77 | J11  M11  P11  J13  I14 | 6 | 4  4  6  4  9 | 295  86  50  38  45 |  |  |  |
|  |  | OXLA_BOTPA |  | 19.02 | M11 | 7 | 9 | 131 |  |  |  |
|  |  | VM1B_BOTIN |  | 19.35 | P11 | 8 | 5 | 65 |  |  |  |
|  |  | VSP1_BUNMU |  | 19.91  20.46  21.45 | L12  G12  C13 | 9 | 21  6  6 | 176  127  127 |  |  |  |
|  |  | VSP1_MACLB |  | 19.91  20.46 | L12  G12 | 10 | 3  3 | 138  43 |  |  |  |
|  |  | VM2IA_BOTIN |  | 19.91 | L12 | 11 | 9 | 124 |  |  |  |
|  |  | VM1B1_BOTBA |  | 19.91 | L12 | 12 | 8 | 62 |  |  |  |
|  |  | VM3_CERRY |  | 20.46 | G12 | 13 | 3 | 44 |  |  |  |
|  |  | VM36A_BOTIN |  | 23.32 | M14 | 14 | 7 | 55 |  |  |  |
|  |  | VM2H1_BOTLA |  | 24.10 | F14 | 15 | 3 | 39 |  |  |  |
|  |  |  |  |  |  |  |  |  |  |  |  |

**SI Uniprot database table.** All Mascot hits found with the Uniprot database in the area where pro- and anticoagulant activity was observed. Table includes information on masses, retention times, well numbers of the nanofractionated toxins, sequence coverage, protein score, toxin class and coagulation activity.
